# Hidden diversity: Transcriptomic and photosynthetic variation among common ‘wild type’ *Chlamydomonas* strains

**DOI:** 10.1111/tpj.70615

**Published:** 2025-12-05

**Authors:** Xin Liu, Olli Virtanen, Sean D. Gallaher, Wojciech J. Nawrocki, Anne G. Glaesener, Sabeeha S. Merchant, Roberta Croce

**Affiliations:** ^1^ Department of Physics and Astronomy, Faculty of Sciences Vrije Universiteit Amsterdam Amsterdam The Netherlands; ^2^ California Institute for Quantitative Biosciences University of California Berkeley California USA

**Keywords:** Chlamydomonas, high light acclimation, NPQ, Photosynthesis, pigments, state transitions

## Abstract

The unicellular green alga *Chlamydomonas reinhardtii* is a widely studied reference organism, particularly in photosynthesis research. It employs photoprotective mechanisms, such as state transitions (ST) and non‐photochemical quenching (NPQ), to cope with rapid light changes. Most widely used strains share a recent common ancestor yet differ by up to ~50 000 nuclear variants—genetic diversity that is often overlooked. Even among ‘wild type’ strains, we document significant phenotypic differences, such as pigment accumulation, and nutrient utilization. To elucidate the basis for this variation, we compared transcriptomes and physiological traits of seven commonly used laboratory strains, including the reference strain and the CLiP mutant library parental strain. Despite identical growth conditions, ~40% of genes were differentially expressed between strains. Most of these differences are attributable to changes that have accrued during laboratory propagation, and adverse conditions may have driven transcriptomic drift. At the physiological level, we catalog the range of strain‐dependent responses related to photosynthesis and high light (HL) acclimation. Specifically, (i) all strains develop NPQ upon HL exposure, but to various degrees, (ii) they show a substantial variation in ST capacity, and (iii) they regulate the composition of the photosynthetic apparatus differently. We find that NPQ levels do not correlate with LHCSR3 expression, suggesting an additional layer of NPQ regulation. STs are constantly activated and independent of growth light intensities. Overall, our findings highlight significant strain‐to‐strain differences in virtually all photosynthetic parameters, emphasizing the importance of careful strain selection in future research endeavors.

## Significance Statement

Laboratory strains of *Chlamydomonas reinhardtii* are widely used as model systems for photosynthesis research, yet they differ genetically and physiologically far more than commonly assumed. By combining transcriptomic and physiological analyses, we reveal major strain‐to‐strain differences in light acclimation and photoprotection. These findings demonstrate that strain background profoundly influences photosynthetic responses and should be carefully considered in the design and interpretation of future studies.

## INTRODUCTION

The unicellular green alga *Chlamydomonas reinhardtii* has been adopted as a model organism for photosynthesis research for decades (Blaby‐Haas & Merchant, 2013; Catalanotti et al., 2013; Johnson & Alric, 2013; Salomé & Merchant, 2019; Yang et al., 2015). In the laboratory, *C. reinhardtii* can grow in either liquid or solid media in three trophic statuses: photoheterotrophic, photoautotrophic, and heterotrophic (Harris, 2009). In nature, *C. reinhardtii* lives in the soil, and it has a flexible metabolism that allows it to adjust to different environmental conditions, including nutrient shortages (Glaesener et al., 2013; Merchant et al., 2020; Saroussi et al., 2017) and light fluctuations (Allahverdiyeva et al., 2015; Erickson et al., 2015).

In natural environments, light energy can fluctuate in both quality and intensity during the diurnal cycle and over shorter time scales, such as those caused by cloud cover or sun flecks. Continuous, moderate light drives photosynthesis with high efficiency. However, when the amount of absorbed light exceeds the capacity of the photosynthetic reactions, harmful chemical species are produced and damage to the photosynthetic apparatus can occur (Erickson et al., 2015; Li et al., 2018). To limit the costly repair from photodamage (Liu et al., 2019; Theis & Schroda, 2016), photosynthetic organisms use a series of strategies on different time scales that permit them to survive and adapt to constantly changing environmental conditions (Erickson et al., 2015; Minagawa & Tokutsu, 2015; Moejes et al., 2017; Rochaix, 2014).

In cyanobacteria, green algae, and plants, the light reactions of photosynthesis involve several multiprotein complexes embedded in the thylakoid membrane: photosystem II (PSII), cytochrome (Cyt.) *b*_6_ *f*, photosystem I (PSI), and ATP synthase. Light energy absorbed via the light‐harvesting complexes (LHCI and LHCII) of PSI and PSII is transferred to the reaction centers (RC) of the photosystems and used for charge separation (Croce & van Amerongen, 2020). In PSII, the oxygen‐evolving complex splits water molecules into oxygen, protons, and electrons. The latter are transferred through the electron transport chain and used for NADPH reduction. The proton gradient created across the thylakoid membrane is used by the ATP synthase to form ATP. ATP and NADPH fuel CO_2_ reduction in the Calvin–Benson cycle. Noticeably, the organization and composition of the thylakoid membrane dynamically depend on light and carbon availability (Polukhina et al., 2016). An increase in light intensity results in a short‐term (light shock) and long‐term (acclimation) response in *C. reinhardtii* cells (Erickson et al., 2015; Niyogi, 2009). The long‐term high‐light (HL) acclimation responses of *C. reinhardtii* have been previously investigated in several studies in various trophic statuses (Bonente et al., 2012; Meagher et al., 2021; Nawrocki et al., 2020; Polukhina et al., 2016; Redekop et al., 2020; Virtanen et al., 2021). At present, we know that HL acclimation involves modification of pigment content (Bonente et al., 2012; Neale & Melis, 1986; Polukhina et al., 2016), photosystem stoichiometry and PSII antenna size (Polukhina et al., 2016). Changes in the expression of the PSII antenna proteins, LHCBM1‐LHCBM9, are one contributing factor (Minagawa & Takahashi, 2004; Natali & Croce, 2015).

In *C. reinhardtii*, there are two main short‐term photoprotective responses: state transitions (ST) and non‐photochemical quenching (NPQ) of excitation energy. STs are triggered by the redox state of the plastoquinone (PQ) pool, which largely reflects excitation imbalances between the two photosystems (Erickson et al., 2015; Minagawa & Tokutsu, 2015; Moejes et al., 2017). When light preferentially excites PSII, increasing the net reduction of the PQ pool, the Stt7 kinase phosphorylates LHCII, which then migrates from PSII to PSI (Depège et al., 2003) in a process known as the State I‐to‐State II transition. When light preferentially excites PSI and the PQ pool becomes more oxidized, the phosphatases (PPH1/PBCP) dephosphorylate LHCII, which then migrates from PSI to PSII in the state II‐to‐state I transition. NPQ, the central process of photoprotection that dissipates excess excitation energy as heat (Ruban, 2016; Ruban, 2018), plays a key role in *C. reinhardtii* photoprotective strategy on a timescale shorter than antenna‐to‐PS ratio adjustments (Erickson et al., 2015). The essential players of NPQ in *C. reinhardtii* are the light‐harvesting complex stress‐related proteins LHCSR1 and LHCSR3 (Peers et al., 2009), which are activated by the low lumenal pH (Bonente et al., 2011; Liguori et al., 2013). The accumulation of LHCSR3, the main player in NPQ, occurs in high light and other stress conditions (Naumann et al., 2007; Polukhina et al., 2016; Terauchi et al., 2010; Zhang et al., 2004). It is generally accepted that a linear correlation between the NPQ level and LHCSR3 content exists (Bonente et al., 2012; Perozeni et al., 2018; Tian et al., 2019). Moreover, NPQ and ST have been shown to have partially overlapping and complementary functions (Roach & Na, 2017). LHCSR3 was suggested to be involved in ST (Allorent et al., 2013), and intriguingly, the mutants lacking Stt7 and consequently deficient in ST, exhibit a much higher NPQ capacity than their respective control cell lines (Bonente et al., 2011; Girolomoni et al., 2019; Nawrocki et al., 2020).

The majority of laboratory strains of *C. reinhardtii* belong to a common lineage that can be traced back to a single zygospore that was isolated in 1945 from soil collected in Amherst, MA. In a prior comparative genomics study, we demonstrated that the genomes of all extant strains in this ‘Amherst’ lineage comprise a mosaic of two haplotypes, which we arbitrarily labeled Haplotype 1 and Haplotype 2 (Gallaher et al., 2015). The two haplotypes are approximately 2% divergent from each other at the nucleotide level (including single nucleotide variants and small insertions/deletions) and are presumed to represent the genetic contributions from the two parental gametes that produced the initial zygospore. Likely as the result of a series of backcrosses early in their history in the laboratory, all of the extant strains in the Amherst lineage carry a single haplotype, called Haplotype 1, over approximately 3/4 of their genomes. The remaining 1/4 of the genome consists of ‘blocks’ (portions of chromosomes) that may be either Haplotypes 1 or 2 depending on the strain. This mosaic distribution of haplotype blocks provides a fingerprint for each strain that is unique to that strain and readily identifiable. Nearly all (98.2%) of the inter‐strain genetic diversity is attributable to the presence of the two haplotypes, such that two strains with different haplotypes may have up to 500 000 variants between them. The small remainder of inter‐strain variants, typically less than 1000 between strains with the same haplotype, is due to mutations that have occurred in the laboratory over the decades since 1945 that these strains have been independently propagated.

Despite their shared lineage, there are frequently significant phenotypic differences between ‘wild type’ strains in the Amherst lineage. These differences include photosynthetic parameters (such as non‐photochemical quenching mechanisms), cell wall presence (Pazour et al., 1995), and nutrient utilization capabilities (Fernández et al., 1989; Gallaher et al., 2015). Many examples of phenotypic variation are readily evident in day‐to‐day experiments, including cell size, pigment content, and trophic growth rates. For example, in an analysis of diel cycle cultures performed on three WT, Amherst‐lineage strains (CC‐124, CC‐1009, and CC‐1690), there were large differences in LHCSR3‐dependent NPQ and state transition capacity (Nawrocki et al., 2021). Given that any two Amherst‐lineage strains may differ at up to 500 000 loci depending on their respective haplotypes, it would be expected that haplotype would have a profound effect on transcription. We sought to examine this directly by producing and comparing transcriptomes for various WT strains grown under identical laboratory conditions, termed here as ‘basal’ conditions. For this analysis, we chose the five strains we had previously shown to be the oldest lines within the Amherst lineage (CC‐124, CC‐125, CC‐1009, CC‐1690, and CC‐1691). Collectively, these five strains represent all the genetic diversity attributable to the two haplotypes. Two additional strains were added based on their importance to the Chlamydomonas research community: strain CC‐4532 is the source of the current reference genome for *C. reinhardtii* (Craig et al., 2023), and CC‐4533 (CMJ030) is the parental strain of the widely used Chlamydomonas Library Project (CLiP) collection of mutant strains (Li et al., 2019). In addition to our basal transcriptome analysis, we performed extensive functional and biochemical studies, comparing the capacity of these seven strains to acclimate to continuous high light (HL). By comparing strains under a standard set of conditions, we sought to reveal differences between the reference WT strains and to potentially explain discrepancies observed between studies performed in different laboratories. We show that while the global strategies of HL acclimation are similar in these seven genetically distinct WT strains, substantial variation in photosynthetic parameters exists, which may contribute to different interpretations and conclusions across laboratories.

## RESULTS

Over the course of our many biochemical studies performed on *C. reinhardtii*, we have observed numerous examples of significant phenotypic differences between different, commonly used strains, despite their recent shared ancestry. These differences can have a confounding effect on experiments, such as comparisons between a WT and a mutant strain, if the researcher does not take this into account in choosing their WT strain. Here we examine a few such phenotypic traits.

### Chl production in the dark‐grown and Cu‐deficient cultures

**Figure 1:**
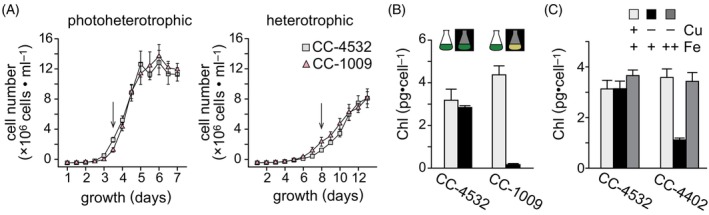
Chl content in the dark and in Cu deficiency is strain‐dependent. (A) Cultures of CC‐4532 and CC‐1009 were pre‐acclimated to either photoheterotrophic (light + acetate) or heterotrophic (dark + acetate) conditions, and grown to stationary phase under the same conditions. Growth was monitored by cell counting. (B) Cells from photoheterotrophic (light gray) and heterotrophic (black) cultures were sampled for Chl analysis at a density of 2–3 × 10^6^ cells·mL^−1^ as indicated by the arrows in panel A. (C) Separately, cultures of CC‐4532 and CC‐4402 were grown photoheterotrophically under three nutrient conditions: replete medium, (20 μM Fe, 2.5 μM Cu, light gray), Cu‐deficient medium (20 μM Fe, no added Cu, black), and Fe‐excess, Cu‐deficient medium (200 μM Fe, no added Cu, dark gray). The mean of three independent cultures is shown for each condition, error bars represent SD.

The conversion of protochlorophyllide to chlorophyllide during Chl biosynthesis is catalyzed in two different ways: in a light‐dependent reaction dependent on a nucleus‐encoded enzyme and / or in a light‐independent reaction dependent on a plastid‐encoded multi‐subunit enzyme (Li & Timko, 1996). Many green algae, including *Chlamydomonas* spp. have both enzymes, and accordingly accumulate Chl proteins, and thus appear green, even when grown heterotrophically in the dark. Nevertheless, some laboratory strains have lost the light‐independent pathway (Cahoon & Timko, 2000). The Chl content of CC‐4532 is only slightly reduced in cells maintained in the dark, 2.8 ± 0.1 pg·cell^−1^, compared to illuminated cells, 3.2 ± 0.5 pg·cell^−1^ (Figure 1B). In contrast, we note that dark‐grown heterotrophic cultures of a different WT strain, CC‐1009, are unable to accumulate Chl, which is measured at 0.2 ± 0.1 pg·cell^−1^ (4%) compared to 4.4 ± 0.4 pg·cell^−1^ for illuminated cells. Despite the inability of CC‐1009 to accumulate Chl in the dark, both strains grow at comparable rates in both the light and the dark (Figure 1A).

Another distinctive pigment phenotype is evident in micronutrient‐deficient cells. Iron‐deficient cells are typically chlorotic because of the Fe requirement of a key enzyme in Chl biosynthesis, namely the aerobic oxidative cyclase, which has a di‐iron active site (Spiller et al., 1982; Tottey et al., 2003). Chl content can therefore be an indicator of Fe status. We noted that the Fe content of certain laboratory strains is more notably dependent on Cu nutrition, which is attributed to the role of a multi‐copper oxidase in high affinity Fe uptake (Herbik et al., 2002; La Fontaine et al., 2002). For instance, Cu‐deficient cultures of CC‐4402 (closely related to CC‐124) are chlorotic with Chl reduced by 69% from 3.6 ± 0.3 pg·cell^−1^ in replete to 1.12 ± 0.07 pg·cell^−1^ in Cu‐deficient cultures (Figure 1C). The reduction is attributed to Fe deficiency because the phenotype can be rescued (3.4 ± 0.4 pg Chl·cell^−1^) by provision of more Fe in the growth medium (Gallaher et al., 2015). On the other hand, CC‐4532 maintains its Chl content at the same Cu status, with 3.1 ± 0.3 pg·cell^−1^ in Cu‐replete, 3.1 ± 0.3 pg·cell^−1^ in Cu‐deficient, and 3.7 ± 0.2 pg·cell−1 in Cu‐deficient/Fe‐excess conditions (Figure 1C).

### Growth rates under different trophic states

**Figure 2:**
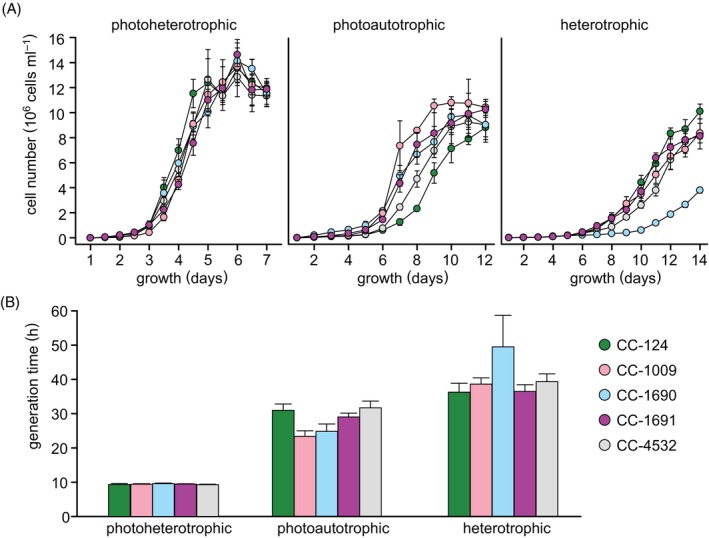
Diversity of growth rates in WT strains. (A) Growth curves are plotted for five WT strains under photoheterotrophic (light + acetate), photoautotrophic (light only), and heterotrophic (dark + acetate) conditions. The mean of three biological replicates is shown. Error bars represent SD. (B) The generation time of each strain in each trophic state was calculated from the logarithmic growth phase. Bars represent the mean value from three replicate determinations. Error bars represent SD.

Most experimentalists grow Chlamydomonas cells in one of three trophic situations, the most common being photoheterotrophic (light + acetate) because of the short generation time (Figure 2). Studies of photosynthetic performance demand photoautotrophic growth, for which conditions can vary with respect to photon flux density (PFD) and CO_2_ supply (air vs. high CO_2_ at 1–5%; Harris, 2009), while studies of respiration demand growth on acetate in the dark. Based on our previous study (Gallaher et al., 2015), we chose five strains from the Amherst lineage that represented the widest range of genetic diversity, and used those to assess the variation in phenotype. Under photoheterotrophic conditions, the most common in laboratories for maintenance and culture, all strains exhibited similar growth rates with generation times ranging from 9.3 to 9.6 h (CC‐124: 9.4 ± 0.3 h, CC‐4532: 9.3 ± 0.2 h, CC‐1690: 9.6 ± 0.2 h, CC‐1009: 9.5 ± 0.2 h, CC‐1691: 9.5 ± 0.1). However, more pronounced differences emerged under the less commonly used single‐trophic growth conditions.

For photoautotrophic growth, the strains clustered into two distinct groups: CC‐124, CC‐1691, and CC‐4532 with similar generation times of approximately 31 h (31.0 ± 1.9, 31.8 ± 2.0, and 29.1 ± 1.1, respectively) versus CC‐1009 and CC‐1690 with faster generation times of ~24 h (24.9 ± 2.2 and 23.5 ± 1.6, respectively) about 7 h faster than the strains in the first group. Strict heterotrophic growth is slow, with strain CC‐1690 showing a long lag phase, but the generation time, 49.6 ± 9.2 h, is only slightly longer (and not statistically different) than that of the other WT strains at 36.4 ± 2.6 h, 39.4 ± 2.3 h, 38.7 ± 1.8 h, and 36.5 ± 2.0 h for strains CC‐124, CC‐4532, CC‐1009, and CC‐1691, respectively. Additionally, there was considerable variation between these strains in terms of cell size and biomass (SI results and Figure S1), and micronutrient content (Figure S2).

The prevalence of so many significant phenotypic differences among these closely related WT cell lines prompted us to undertake a more extensive molecular phenotyping with respect to pathways, such as photosynthesis, and associated experimental approaches, such as transcriptomics, that are of general interest to the Chlamydomonas research community.

### Differences in basal transcriptomes between WT strains

**Figure 3:**
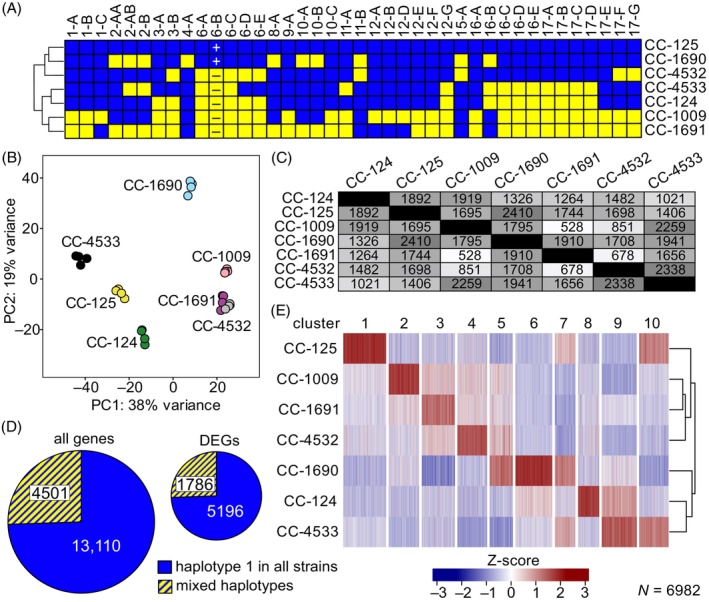
Genetic and transcriptomic variation between WT strains. (A) The mosaic distribution of haplotype 1 (blue) and haplotype 2 (yellow) blocks for the seven strains used in this study. Haplotype 2 is ~2% divergent from haplotype 1. See Gallaher et al. and Craig et al. for additional information on haplotypes (Craig et al., 2023; Gallaher et al., 2015). The ‘+’ and ‘–’ in Block 6‐B indicates the *C. reinhardtii* mating locus. (B) A principal component analysis of the 1000 most divergent genes in the basal transcriptomes of the seven indicated strains. Four biological replicates for each strain are plotted. The contributions of the first and second principal components are indicated in the axis labels. (C) Transcript abundances of all nucleus‐encoded genes for seven strains were determined by RNA‐Seq analysis. The number of pairwise differentially expressed genes (DEGs) between each of the seven strains is shown as a table. See Methods for criteria used to call DEGs. Data on the DEGs, including gene IDs, fold‐change, and haplotype, may be found in SI Dataset S1. (D) On the left, a pie chart of all nuclear genes (*n* = 17 611) classified as being Haplotype 1 in all seven strains (blue), or a mixture of both haplotypes between strains (blue/yellow stripes). On the right, a proportionally scaled pie chart shows the subset of DEGs (*n* = 6982) classified with the same criteria. For both pie charts, the blue/yellow portion represents 25.6% of the total. (E) Transcript abundances for the 6982 genes found to be DEGs between at least two strains were Z‐score normalized and sorted by *k*‐means clustering into 10 groups. Functional enrichment for each of the gene clusters is provided in SI Dataset S2.

Given the phenotypic diversity between the closely related laboratory strains of *C. reinhardtii*, we sought to identify differences in RNA abundances under standard laboratory growth conditions (subsequently called ‘basal’) that could help clarify the contribution of genetic variation to molecular phenotypes. Five strains were chosen as the most ancestral strains in the Amherst lineage (CC‐124, CC‐125, CC‐1009, CC‐1690, and CC‐1691), and an additional two for their importance to the Chlamydomonas community (CC‐4532 and CC‐4533). Based on their distribution of haplotype blocks (Figure 3A), all genetic variation attributable to the two haplotypes is represented by these strains, which accounts for >98% of the total genetic variation among the WT Amherst‐lineage strains (Gallaher et al., 2015). To produce basal transcriptomes, each strain was grown in four biological replicate cultures in photoheterotrophic conditions under continuous light in nutrient‐replete medium, and mRNA was collected during the mid‐log growth phase (~2 × 10^6^ cells·mL^−1^) for analysis by RNA‐Seq. Principal component analysis (PCA) performed on the 1000 genes with the greatest variance confirmed that the four replicates were highly reproducible, and that each strain was readily distinguishable from the others, with CC‐1690 and CC‐4533 being the most unique (Figure 3B). Curiously, the PCA shows that CC‐4532 and CC‐1691 were the most similar at the transcriptome level despite being highly divergent at the haplotype level (Figure 3A). To examine the transcriptomic differences further, we identified differentially expressed genes (DEGs) between each pair of strains and observed between 528 (3.0%) and 2410 (13.6%) inter‐strain DEGs (Figure 3C). Since all seven strains were grown under identical laboratory conditions, it was surprising that 6982 genes, representing 39.5% of all genes, were differentially expressed in this set of strains. It was anticipated that the different haplotype block distributions between strains would be a proxy for the number of DEGs. In contrast, the haplotypes turned out to be a poor predictor of the number of DEGs between strains: for example, CC‐1009 and CC‐4532 have very different haplotypes (Figure 3A) but have some of the fewest DEGs (Figure 3C).

Having found an unexpectedly poor correlation between haplotype and DEGs, we wished to examine this more directly. If mixed‐haplotype genes (i.e., a gene that is Haplotype 1 in one strain and Haplotype 2 in the other) are more likely to be differentially expressed between those strains, one would expect an enrichment (i.e., a higher percentage) of mixed‐haplotype genes to be found among the 6982 genes that are differentially expressed. In contrast, we observed no meaningful enrichment (*p*‐value = 0.53). Remarkably, the same percentage of mixed‐haplotype genes, 25.6%, was observed in both the DEG population and in all genes (Figure 3D). This finding is consistent with there being no significant contribution from the different haplotypes to differences in transcription.

Next, we sorted the 6982 DEGs into 10 bins by *k*‐means clustering based on each gene's pattern of expression (Figure 3E). By plotting the expression as a heatmap, patterns were observable in which groups of several hundred genes were expressed at a much higher level in one or two strains. To identify shared functionality, we performed gene ontology (GO) enrichment on each of the clusters (SI Dataset S2). While not all clusters had statistically significant enrichment of GO terms, a few interesting patterns were revealed. Cluster 1, which is significantly upregulated in strain CC‐125, includes many genes annotated as being involved in the response to freezing and ice binding. Cluster 3, with genes largely upregulated in strain CC‐1691, had significant enrichment of cell wall‐related genes, and Cluster 9, primarily upregulated in strain CC‐4533, was enriched for genes involved in DNA repair.

**Figure 4:**
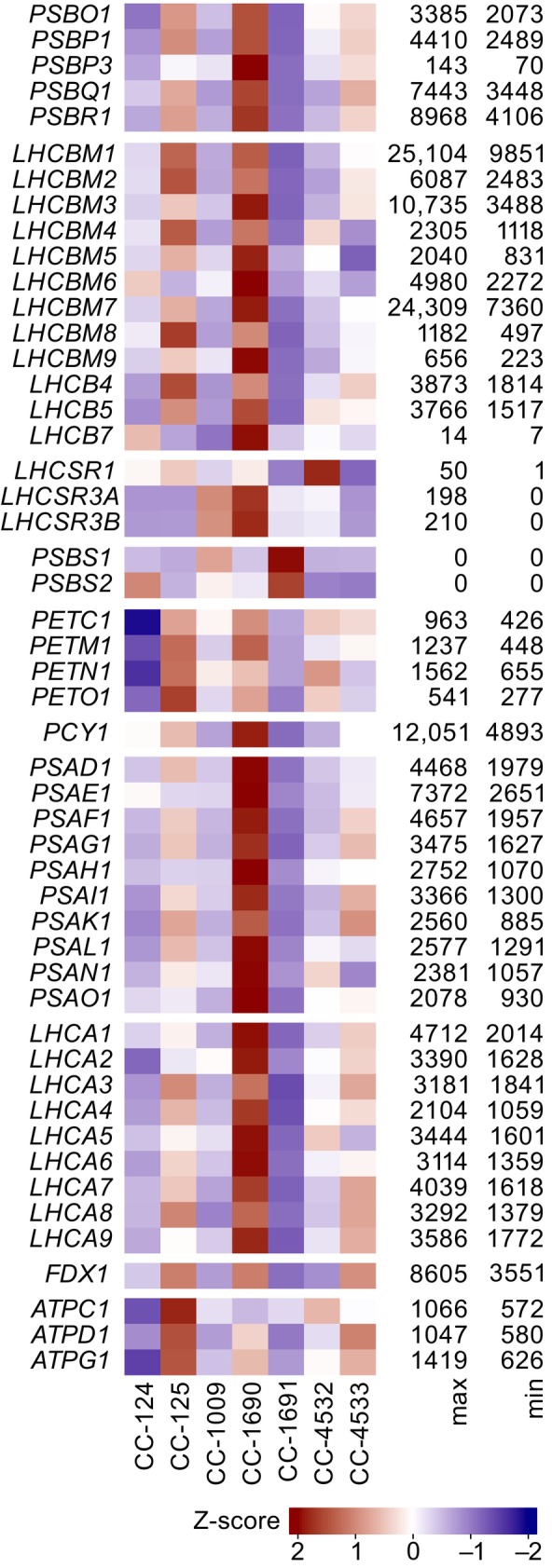
Transcriptomic analysis of the photosynthetic apparatus. Transcript abundances were calculated for the seven strains in terms of FPKMs for nucleus‐encoded genes that encode components of the photosynthetic apparatus. FPKMs were Z‐score normalized and plotted as a heatmap. The maximum and minimum FPKMs for each gene are shown on the right.

Previously, we had identified phenotypic differences between several WT strains in a number of photosynthesis‐related parameters (Gallaher et al., 2015; Nawrocki et al., 2021). To examine this directly in these seven strains, we plotted the expression patterns of genes involved in photosynthesis and the biosynthesis of several pigments. For most genes encoding components of PSI and PSII, as well as LHCI and LHCII, strain CC‐1690 consistently had the highest expression levels (Figure 4). There was greater uniformity for components of the Cyt. *b*_6_ *f* complex, and strain CC‐125 had the highest expression of components of the ATP synthase. Given the modest light levels to which these cultures were subjected (~60 μmol photons m^−2^·s^−1^), there was relatively low expression of genes involved in NPQ. For genes involved in Chl biosynthesis, the range of expression between strains was generally lower, with strain CC‐4532 expressing most genes at the highest level (Figure S3). Expression of genes involved in the biosynthesis of carotenoids (Cars) and lutein (Figure S4) was mostly quite low, with strain CC‐4532, expressing the highest levels of most carotenoid biosynthesis genes.

### Variations in pigment composition among WT strains

Next, we examined how these differences at the level of the transcriptome could affect photosynthetic parameters, such as pigment composition and acclimation to HL. Cultures of the same seven strains from the transcriptomic analysis were grown photoheterotrophically (light + acetate) to exponential phase in two typically used light intensities (low light, LL: 20 μmol photons m^−2^·s^−1^, and moderate light, ML: 80 μmol photons m^−2^·s^−1^). Cells grown in ML were subsequently collected by centrifugation and resuspended in acetate‐free medium to transition to photoautotrophic growth under continuous high light (HL: 500 μmol photons m^−2^·s^−1^). It was previously shown that the doubling time of the CC‐124 strain in HL in photoautotrophic conditions is approximately 24 h (Polukhina et al., 2016). We thus considered that acclimation to HL is completed after 6 days (144 h) with regular dilutions to maintain cells in growth phase, and that this corresponds to approximately six generations.

**Figure 5:**
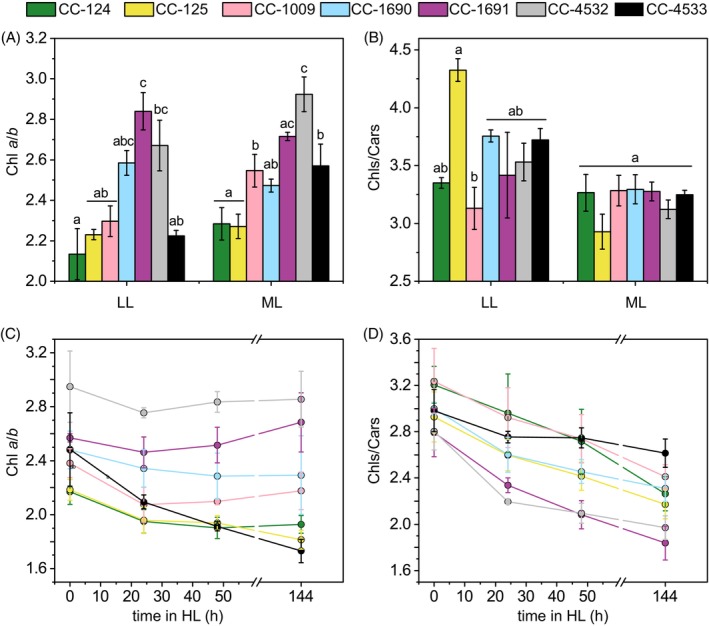
Pigment composition varies by strain. (A, C) Chlorophyll *a*/*b* ratio and (B, D) chlorophyll/carotenoids ratio. Panels A and B show values for cultures in photoheterotrophic conditions (mean ± SD, n = 2 in LL and n = 3 in ML) after 4 days. Panels C and D show the kinetics of the shift from ML to HL (mean ± SD, n = 4 for 0, 24, 48 h HL and n = 6 for 144 h HL). Identical letters above the bars in A and B indicate a lack of statistically significant difference (p < 0.05) between strains, examined via one‐way ANOVA independently for both light intensities.

Under photoheterotrophic conditions, large differences in the Chl *a*/*b* ratio exist between genotypes. These differences are independent of the growth light intensity (LL or ML), with the highest value (~2.9) observed in CC‐1691 and CC‐4532 and the lowest in CC‐124 and CC‐125 (~2.1 and ~ 2.2, respectively) (Figure 5A). Conversely, the Chls/Cars ratios were similar in all strains under ML conditions, except for CC‐125 in LL. The ratio was generally higher in LL than in ML conditions (Figure 5B).

Upon HL exposure, the Chl *a*/*b* ratio of most strains decreased, particularly during the first 2 days of acclimation (Figure 5C). Interestingly, the ratio remained stable in CC‐4532 and even showed a slight increase in CC‐1691. In contrast, CC‐125 and CC‐4533 were the only strains to show a decline in the ratio between 48 and 144 h of acclimation, suggesting that some acclimation processes might proceed more slowly in these strains between these two timepoints.

In all strains, the Chls/Cars ratio decreased under HL by about 30% with some variation (Figure 5D). To detect possible changes in the Car composition, we separated the pigments by liquid chromatography. We observed few differences between LL and ML in Car content, but upon 6 days of HL, the relative amount of lutein and/or zeaxanthin increased dramatically in all strains (Table 1). Strain CC‐4532 produced the highest levels of lutein and Car when grown in ML, which comports nicely with the transcriptomic analysis described above (Figure S4).

**Table 1:** Carotenoid composition in WT strains

Light conditions	Strains	Neo and Lor	Vio	Anth	Lut	Carotenes
Low light	CC‐124	7.1 ± 0.3	8.3 ± 0.6	0.4 ± 0.1	6.5 ± 0.5	7.2 ± 0.4
	CC‐125	7.9 ± 2.4	3.9 ± 0.0	1.2 ± 0.0	6.1 ± 0.3	5.1 ± 0.3
	CC‐1009	10.7 ± 1.3	5.9 ± 0.8	0.3 ± 0.0	6.2 ± 0.5	8.0 ± 0.7
	CC‐1690	8.1 ± 1.8	6.5 ± 0.1	0.7 ± 0.1	5.0 ± 0.4	6.5 ± 0.0
	CC‐1691	8.6 ± 0.6	6.8 ± 0.3	0.6 ± 0.1	6.2 ± 0.3	8.1 ± 0.9
	CC‐4532	7.6 ± 0.9	6.7 ± 0.2	0.8 ± 0.3	7.4 ± 1.8	7.7 ± 1.0
	CC‐4533	8.3 ± 0.9	6.7 ± 0.4	0.5 ± 0.0	5.3 ± 0.1	5.7 ± 0.5
Moderate light	CC‐124	6.9 ± 1.0	8.4 ± 0.5	0.4 ± 0.3	6.5 ± 0.7	5.7 ± 1.2
	CC‐125	11.2 ± 1.7	7.2 ± 1.4	0.4 ± 0.2	7.7 ± 1.1	6.4 ± 0.3
	CC‐1009	7.0 ± 0.7	6.0 ± 0.5	0.7 ± 0.5	6.1 ± 0.3	6.0 ± 1.1
	CC‐1690	9.2 ± 0.5	9.2 ± 1.0	0.6 ± 0.3	6.6 ± 0.5	6.4 ± 0.9
	CC‐1691	6.4 ± 0.4	6.6 ± 0.5	0.4 ± 0.1	6.4 ± 0.4	5.9 ± 0.6
	CC‐4532	7.0 ± 1.8	7.0 ± 1.6	0.3 ± 0.1	9.9 ± 1.5	7.4 ± 1.8
	CC‐4533	6.9 ± 0.4	6.6 ± 0.3	0.3 ± 0.1	5.6 ± 0.0	5.6 ± 0.9

Light conditions	Strains	Neo and Lor	Vio	Anth	Lut and Zea	Carotenes
High light (144 h)	CC‐124	10.0 ± 0.5	6.0 ± 1.2	1.7 ± 0.4	22.4 ± 2.3	5.5 ± 0.7
	CC‐125	10.5 ± 0.7	4.2 ± 0.0	2.2 ± 0.1	22.8 ± 0.9	5.5 ± 0.3
	CC‐1009	8.8 ± 0.9	5.9 ± 1.3	1.1 ± 0.1	16.6 ± 0.6	5.3 ± 0.4
	CC‐1690	10.0 ± 0.9	7.6 ± 0.8	1.4 ± 0.4	15.9 ± 0.1	5.0 ± 0.1
	CC‐1691	6.4 ± 1.3	7.0 ± 1.1	2.1 ± 0.3	20.6 ± 0.3	9.6 ± 0.5
	CC‐4532	6.2 ± 0.4	6.0 ± 0.4	4.4 ± 0.2	26.7 ± 0.9	8.0 ± 0.5
	CC‐4533	9.7 ± 1.6	5.2 ± 0.5	1.5 ± 0.3	16.8 ± 0.3	2.4 ± 0.2

Data shown are normalized to 100 Chl molecules. Each value is the mean ± SD, n = 2 (LL), n = 3 (ML), and n = 2 (144 h HL) from biological repetitions each with three technical replicates. Lut and Zeaxanthin cannot be perfectly separated and their content in HL is therefore summed.

### Variations in PSII acclimation among WT strains

**Figure 6:**
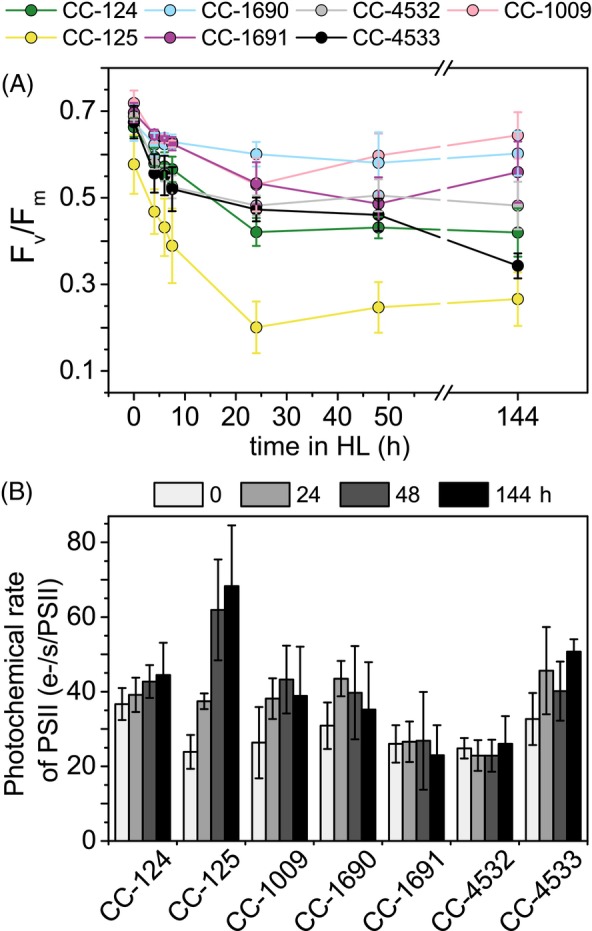
*F*_v_/*F*_m_ and PSII antenna size during HL acclimation. (A) Maximum quantum efficiency of PSII photochemistry (*F*_v_/*F*_m_), and (B) functional antenna size of PSII. Data shown (A) are mean ± SD, n = 3 for 4, 6, 7.5 h HL; and n = 7 for 0, 24, 48, 144 h HL. Data shown in (B) are mean ± SD, n = 5–6 biological replicates.

To determine PSII functionality under HL conditions, we measured the *F*_v_/*F*_m_ value, a commonly used indicator of the photochemical capacity of PSII. As shown in Figure 6A, *F*_v_/*F*_m_ ranged from 0.58 (CC‐125) to 0.72 (CC‐1009) before HL treatment and decreased in all strains in response to the HL exposure. However, while the decrease was moderate in CC‐1009 and CC‐1690, it was much larger in CC‐125, reaching a value of 0.27 after 6 days in HL. Overall, two main trends were observed across the strains. In some strains (CC‐124, CC‐125, CC‐1690, CC‐4532), *F*_v_/*F*_m_ decreased almost as a monoexponential decay. In others (CC‐1009, CC‐1691), *F*_v_/*F*_m_ initially dropped but mostly recovered over the 6 days of acclimation. Only CC‐4533 showed a continued decrease in *F*_v_/*F*_m_ throughout the HL treatment. These data indicate considerable variation in the acclimation capacity and/or strategies of the strains.

A typical response to different light conditions is a change in the antenna size of the photosystems (Polukhina et al., 2016; Wientjes et al., 2013). To determine whether this response varies among WT strains, the functional antenna size of PSII was measured during HL acclimation (Figure 6B). Differences between the strains were observed even before HL treatment. For example, the PSII antenna size of CC‐124 was 30% larger than that of CC‐125. In most strains, the functional antenna size increased during the first 2 days in HL, resulting in an increase of the PSII cross‐section ranging from 16.5% (CC‐124) to 259% (CC‐125), which is likely due to a decrease in functional PSII core. After 6 days of HL, the PSII absorption cross‐section either increased even further in some strains (CC‐124, CC‐125, CC‐4533) or remained at the level reached after 2‐day acclimation (CC‐1009, CC‐1690). In contrast, CC‐1691 and CC‐4532 maintained their initial PSII antenna size throughout the HL treatment, showing no measurable response.

### The PSI:PSII Ratio depends on the genetic background

Since the photosystems' stoichiometry is essential for regulating the photosynthetic electron transport chain, PSI:PSII ratios were measured. Two methods were used: (i) electrochromic shift (ECS) signal, which is based on the charge separation in the reaction centers *in vivo* and provides the ratio between functional complexes, and (ii) immunoblots against specific subunits of PSI and PSII, which provide the overall relative protein ratios.

**Figure 7:**
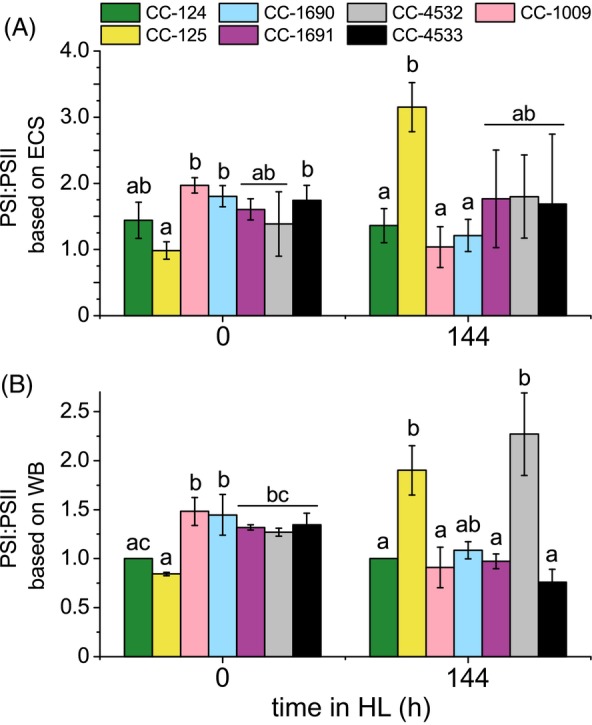
PSI:PSII ratios before and after 6 days in HL. (A) Functional PSI:PSII reaction center ratio measured *in vivo* using the electrochromic shift probe. Data shown are mean ± SD, n = 3 biological repetitions. (B) Relative PSI:PSII ratio according to immunoblot quantification of PsaA:CP47 protein signals. Data shown were normalized to CC‐124 and are mean ± SD, n = 2 biological repetitions. Identical letters above the bars indicate a lack of statistically significant difference (p < 0.05) between strains, examined via one‐way ANOVA independently for each timepoint.

The functional PSI:PSII ratio was different among the WTs even prior to HL exposure, with CC‐125 exhibiting the lowest ratio (~1), while all other strains showed values in the 1.4–2 range (Figure 7A). A very similar trend was observed when the ratio was probed biochemically through immunoblotting, using PsaA and CP47 as representative proteins for PSI and PSII, respectively (Figure 7B and Figure S5).

Interestingly, after 6 days in HL, the functional ratio was similar to pre‐HL levels in four of the strains (CC‐124, CC‐1691, CC‐4532, CC‐4533), despite differences in their acclimation dynamics during the initial 48 h (Figure S6a) and large variations in *F*_v_/*F*_m_ (Figure 6A). Three of these strains also exhibited a decrease in the PSI:PSII ratio based on protein levels (Figure 7B). This suggests a shared characteristic between these strains: a re‐equilibration of the photosystem ratio following the initial stress response, likely to maintain balanced electron flow. In contrast, the three other strains, which did not return to their original functional PSI:PSII ratios, showed two types of responses. CC‐1009 and CC‐1690 exhibited a reduction in both functional and protein‐based PSI:PSII ratios after the HL acclimation. CC‐125, on the other hand, showed a drastic increase in the PSI:PSII ratio as measured via both functional (320%) and protein (192%) analysis.

The increases in functional PSI:PSII ratios observed during the first 2 days of HL (Figure S6a) are not unexpected. During this early phase, PSII is more susceptible to photodamage, particularly before full NPQ capacity is activated. This results in an excess of damaged PSIIs, which reduces the number of functional PSII complexes and leads to a higher PSI:PSII ratio measured via ECS. In contrast, the immunoblot results reflect only total protein abundance and are thus less influenced by the PSII functional state (Figure S6b).

### ST capacity differs between strains, and it is preserved in HL

STs are a process of antenna redistribution between PSII and PSI. In land plants, STs are most relevant in LL conditions, when the PS antenna changes have a proportional effect on electron transfer. In green algae, this process remains fully operational even at peak light mid‐day (Nawrocki et al., 2020), and is considered to be an integral part of photoprotection. To examine the capacity of the WT strains to perform ST, we measured the maximal fluorescence in states I and II, which reflects changes at the PSII level. The cells were treated with 10 μM DCMU under low‐intensity red light to induce State I. State II was instead induced in a light‐independent way by adding glucose (20 mM) and glucose oxidase to bring the cells to anoxia (Wollman & Delepelaire, 1984).

**Figure 8:**
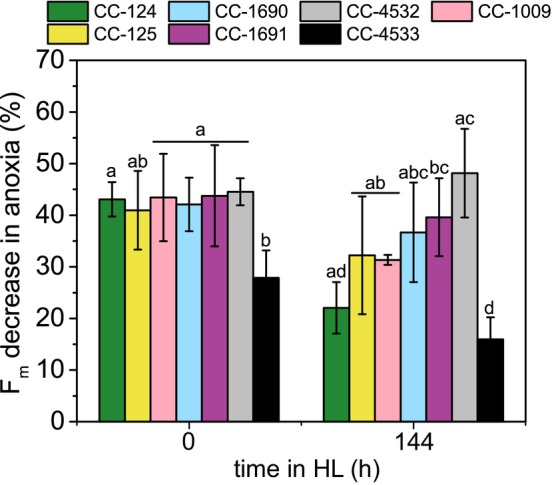
ST capacity upon HL acclimation. The decrease in fluorescence yield between State I (induced by DCMU and LL illumination) and State II (anoxia in darkness) is plotted. Data shown are mean ± SD, n = 5 for 0 and 144 h; n = 4 for 24 and 48 h. Identical letters above the bars indicate a lack of statistically significant difference (p < 0.05) between strains, examined via one‐way ANOVA independently for both timepoints.

At time zero of HL acclimation, in most of the strains the maximal PSII fluorescence decreased during the State I‐to‐II transition by approximately 40–45%, except for CC‐4533 for which the ST amplitude was only ~25% (Figure 8 and Figure S7). Strikingly, after HL acclimation (6 days of HL), the capacity of ST in different strains varied dramatically: it decreased in CC‐124, CC‐1009, CC‐1690, and CC‐4533 and remained the same after a transient increase in CC‐125, CC‐1691, and CC‐4532 (Figure S7). After 6 days of HL, the change in PSII fluorescence during the State I to II transition ranged from 15 to 55%, depending on the strain. Interestingly, the pre‐acclimation to autotrophy increased the ST amplitude in most of the strains (all but CC‐125 and CC‐4532) in ML (Figure S7). This is not surprising, as at least in CC‐124, the LHCII abundance, that is, substrate availability for Stt7, increases significantly during the transition to autotrophy (Polukhina et al., 2016).

### NPQ capacity and LHCSR3 abundance do not correlate between strains

**Figure 9:**
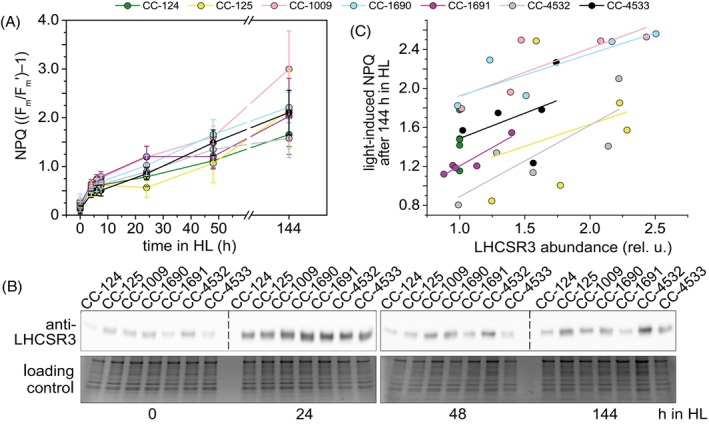
NPQ capacity under HL. (A) Cultures of the indicated strains were grown photoheterotrophically in ML. At t = 0, cultures were transitioned to photoautotrophic growth in HL, and assayed for NPQ capacity. The maximal NPQ capacity is plotted as a function of HL acclimation time. Data shown are mean ± SD, n = 7 biological replicates. (B) The kinetics of the LHCSR3 accumulation throughout the HL treatment as detected by immunoblotting. (C) The correlation between light‐inducible NPQ and LHCSR3 after 6 days of HL acclimation. LHCSR3 abundances are normalized to the LHCSR3 abundance in CC‐124 in the respective immunoblots. Each datapoint describes the ratio in a single biological replicate. The lines describe a best fit to a linear function between biological replicates, performed with least‐squared sum fitting performed separately for each strain with all datapoints belonging to the same strain. Data shown were normalized to the LHCSR3 content in CC‐124 and are mean ± SD, n = 5 biological replicates.

Next, we compared the ability of the WT strains to perform NPQ. In *C. reinhardtii*, NPQ is not a constitutive photoprotection feature, and its capacity depends on a prior HL exposure (Peers et al., 2009; Polukhina et al., 2016). As shown in Figure 9A, none of the strains was able to perform NPQ in ML in photoheterotrophic conditions, but within 24 h of HL exposure, they all developed NPQ capacity identical to that of cells grown photoautotrophically in ML (Figure S8), and both types of cells had similar NPQ capacity after 6 days in HL. However, the capacity to perform NPQ increased during HL treatment with different kinetics for the different strains, with CC‐125 exhibiting the longest lag phase in NPQ development. The steady‐state level of NPQ also showed some differences between strains.

As NPQ in *C. reinhardtii* has been shown to depend on the proteins LHCSR1 and LHCSR3 (Peers et al., 2009), we measured their content in the cells (Figure 9B and Figure S9). After 6 days of HL, the level of LHCSR3 differed between strains, with CC‐125 and CC‐4532 showing the highest content and CC‐124 and CC‐1691 the lowest (Figure 9B). Interestingly, there was no correlation between the steady‐state NPQ level and the LHCSR3 accumulation (Figure 9C), suggesting that some other factors play an essential role in modulating the NPQ capacity in addition to LHCSR3. In the SI materials, we examine the role of the lumenal pH by inducing NPQ with acid, and we calculate a theoretical NPQ based on the maximum *F*_v_/*F*_m_. Neither approach identified a correlation with LHCSR3 abundance (Figures S10 and S11), which hints at a more complex regulation.

## DISCUSSION

In this work, we have compared the photosynthetic parameters of seven laboratory reference strains of the green alga *C. reinhardtii*: CC‐124 (mt–, 137c–), CC‐125 (mt+, 137c+), CC‐1009 (mt–, UTEX89), CC‐1690 (mt+, 21gr+), CC‐1691 (mt–, 6145), CC‐4532 (mt–, the genome reference strain), and CC‐4533 (mt–, parental strain of the CLiP mutant library). The choice of these strains was based on their distant backgrounds, which affect phenotypes and gene expression (Gallaher et al., 2015), as also supported by the large variation in pigment compositions, cell size, and growth rates observed in this work in LL and ML. Here, we have assessed a range of functional and biochemical parameters to obtain quantitative information about the extent to which the acclimation capacity to HL is genetically controlled.

### Source of large‐scale differences in basal transcription

Even though these seven WT strains were grown under identical conditions, a surprisingly large percentage of genes, 39.5%, was differentially expressed by two‐fold or more. From each strain's unique haplotype pattern (Figure 3A) and our prior study (Gallaher et al., 2015), these strains have between 100 000 and 500 000 nt variants between them that were inherited from the genetically divergent gametes that produced the Amherst lineage. It would be reasonable to expect that this degree of genetic variation would have a profound effect on the transcriptome. In contrast, there did not appear to be any observable, large‐scale differences in RNA abundances that could be attributed to differences in haplotype (Figure 3). This was most clearly demonstrated by the observation that the proportion of mixed‐haplotype genes among the DEGs is the same as the proportion of mixed‐haplotype genes among all genes (Figure 3D). If differences in RNA abundances cannot be attributed to a strain's inheritance of the two haplotypes, the next most likely explanation is that these differences have arisen while the strains were maintained in laboratories in the period since 1945. Further, many of these observed differences in basal transcription may not be entirely random. Strain CC‐125 was found to have significantly higher expression of genes involved in the response to freezing and ice binding (Figure 3E and SI Dataset S2). It is speculative, but not unreasonable, to imagine that this strain was once maintained in conditions that were too cold (a common microbiology practice), and this put selective pressure on the strain to increase expression of cold‐protective genes. Similarly, strain CC‐4533, which is the parental strain of the CLiP mutant library, had higher expression of genes involved in DNA repair. One of the strains ancestral to CC‐4533 was subjected to *N*‐Methyl‐*N′*‐nitro‐*N*‐nitrosoguanidine mutagenesis (Davies & Plaskitt, 1971). Perhaps this or other DNA‐damaging stressors created selective pressure for increased expression of DNA repair genes. Collectively, these observations support the idea that gene expression in even closely related WT strains may diverge in profound ways over time under laboratory conditions.

These findings offer a caution with respect to the interpretation of lists of DEGs when strain background has not been taken into consideration. Ideally, all experiments would be conducted exclusively in isogenic strains. But when this is not possible, or when comparing experiments performed in different groups with different strains, it may be beneficial to consider the range of inter‐strain variation in transcript abundance observed in this data set. To that end, we provide normalized transcript abundances, as FPKMs, for all strains and their inter‐strain variance (SI Dataset S3) and fold changes for all pairs of strains (SI Dataset S4) as a resource for the community. For example, ferredoxin 1 (*FDX1*, Cre14.g626700) and *LHCBM1* (Cre01.g066917) have extremely high inter‐strain variance, differing by up to ~2.5‐fold between strains. In contrast, prolyl 4‐hydroxylase 2 (*PFH2*, Cre10.g428500) and dihydrolipoamide acetyltransferase 3 (*DLA3*, Cre06.g252550) have much more modest inter‐strain variances, differing by 1.1‐fold and 1.2‐fold, respectively. Cross‐referencing inter‐strain comparisons with the SI datasets included with this work may help mitigate the danger of misinterpreting differences in expression.

### HL acclimation in *C. reinhardtii*

During the transition from ML to HL, *C. reinhardtii* cells modify their photosynthetic machinery to cope with excess light irradiation and achieve a HL acclimated state. It is expected that the cells acclimate to HL by changing the composition and organization of the photosynthetic apparatus, and by inducing photoprotective responses (NPQ and ST) to minimize photodamage (Erickson et al., 2015; Minagawa & Tokutsu, 2015).

HL acclimation for cells grown photoautotrophically has been reported to influence the pigment composition by: (i) strongly decreasing the Chl content per cell (Bonente et al., 2012; Neale & Melis, 1986; Polukhina et al., 2016; Virtanen et al., 2021), and (ii) significantly decreasing the Chls/Cars ratio without changing the Chl *a*/*b* ratio (Bonente et al., 2012; Cazzaniga et al., 2020; Polukhina et al., 2016). Our work shows that the Chls/Cars ratio decreases in all WTs during HL acclimation in a similar fashion (Figure 5D). However, at variance with the results from the above‐mentioned works, we also observed variations in the Chl *a*/*b* ratio after 6 days of HL exposure (acclimated state) in most strains. Surprisingly, the changes are in different directions, increasing in two strains but decreasing in the others (Figure 5C). Part of these strain‐dependent differences is due to simultaneous acclimation to autotrophy, as the kinetics of Chl *a*/*b* ratios show only minor adjustments in HL when the cells are pre‐acclimated to photoautotrophy, and only in CC‐4532 and CC‐4533 does the ratio decrease during the HL treatments (Figure S12).

The variation of Chl *a*/*b* might result from the regulation of the antenna size, due to the exclusive binding of Chl *b* to the outer antenna, or from a change in PSI/PSII ratio since the antenna of PSII contains more Chl *b* than that of PSI (Drop et al., 2011; Drop et al., 2014). In agreement with this, the antenna size of PSII was shown to correlate with changes in Chl *a*/*b* ratio (Figures 5C and 6B), increasing in all strains that showed a decrease in Chl *a*/*b* ratio, and remaining constant in CC‐1691 and CC‐4532. The latter two strains seem to function similarly to a cell wall‐deficient strain, *cw15*, which also did not exhibit a change in Chl *a*/*b* ratio nor significant changes in PSII antenna (Bonente et al., 2012). Notably, antenna modulation in HL was most pronounced in strains that exhibited the largest differences between photoheterotrophically and photoautotrophically grown cells prior to HL treatment (Figure 7B and Figure S13), suggesting that trophic status has a stronger effect on antenna size than does light intensity. However, this response also shows a high degree of strain dependency, as no major changes were observed in fully photoautotrophic CC‐1691 and CC‐4532 during the HL exposure (Figure S13). Regardless, previous studies showed that upon LL to HL shift, the cells endure a severe reduction in both PSI and PSII abundance, while the PSI/PSII ratio is not affected (Bonente et al., 2012; Polukhina et al., 2016; Virtanen et al., 2021). Hence, our functional data and immunoblot analyses (Figure 7) likely reflect the combined acclimation response to both autotrophic growth conditions and HL with a high degree of strain dependence.

### State transitions remain active in HL‐grown *C. reinhardtii*

In land plants and green algae, state transitions (ST) were thought to be mainly relevant in LL (Goldschmidt‐Clermont & Bassi, 2015), when the PS antenna changes proportionally affect electron transfer due to the distinct absorption spectra of the two photosystems. However, it was also shown that HL‐acclimated plants are capable of ST (Wientjes et al., 2013). In *C. reinhardtii*, STs were shown to be active in LL (Delosme et al., 1996; Nagy et al., 2014; Nawrocki et al., 2016; Ünlü et al., 2014) and diel cycles (Nawrocki et al., 2020), but were proposed to have a role also in HL by reducing the antenna size of PSII at the onset of the HL, before LHCSR3 accumulates in the cells (Allorent et al., 2013). This was not observed under strong illumination in LL‐grown strains that retain the capacity to do ST, as the cell remained in State I (Nawrocki et al., 2021). In the present work, we have measured maximal ST amplitude during acclimation to continuous HL to understand whether ST capacity is maintained in these conditions (Figure 8 and Figure S7). Indeed, all WTs are capable of ST after 6 days in HL, implying that ST can operate despite long‐term HL exposure. Our recent work performed in standard laboratory conditions also showed that the ST capacity of the selected WTs is preserved throughout the diel cycle, even at HL intensity (Nawrocki et al., 2020). Furthermore, ST and NPQ have been shown to be fundamentally linked and partly compensatory to one another in *C*. *reinhardtii* (Steen et al., 2022). We speculate that despite the apparent preference of the cells to remain in State I under HL, they retain the capacity to transfer the antenna to PSI upon changes in the environmental conditions. It is likely an adaptation to fluctuations in light and oxygen availability throughout the day.

As ST are a fundamental component of *C. reinhardtii* photosynthesis regardless of light intensity, differences in ST capacity reflect the genetic differences between strains (Figure 8). Moreover, ST capacities in the WTs during HL acclimation showed a decrease in most of the strains compared to LL (Figure 8), also in the cases when the PSII antenna size and thus the number of LHCII per PSII core increases (Figure 6B). This effect can be explained by a net reduction of the PQ pool when photosynthesis is saturated at the level of PQH_2_ oxidation compared to growth in LL (Sacksteder & Kramer, 2000), since the trigger of ST from state I to II transition is the reduction of the PQ pool, via either chlororespiration or PSII photochemistry (Wollman, 2001).

### The genetic background has a profound influence on NPQ

NPQ development upon HL exposure is a photoprotective strategy common to all analyzed strains, although the extent of NPQ induction varies among them (Figure 9A). This variation can be attributed to differences in genetic background (Gallaher et al., 2015; Nawrocki et al., 2020), which have previously been shown to affect photosynthetic phenotypes.

In *C. reinhardtii*, LHCSR3 is essential for NPQ (Peers et al., 2009) and several studies have demonstrated a linear correlation between LHCSR3 abundance and NPQ levels within individual strains (Bonente et al., 2012; Perozeni et al., 2018; Tian et al., 2019). However, the correlations between LHCSR3 and NPQ (or NPQ_(T)_) are no longer observed when comparing strains (Figures S10 and S11). One possible explanation is that the luminal pH, known to regulate LHCSR3 activation, may differ between strains. To test this hypothesis, we induced NPQ using acetic acid, a method previously shown to trigger stable and reversible quenching (Tian et al., 2019). Yet, as in the case of HL‐induced NPQ (Figure 9 and Figure S11), no correlation was observed between the NPQ level and the LHCSR3 content across the WTs strains (Figure S10). These results show that in addition to LHCSR3 accumulation and luminal acidification, other factors contribute to the modulation of NPQ capacity in *C. reinhardtii*, highlighting the profound influence of genetic background on the photosynthetic traits in this alga.

### A cautionary note

Here, we have demonstrated numerous ways in which commonly used ‘wild type’ strains of *C. reinhardtii* exhibit dramatically different phenotypic characteristics. This phenomenon can have an unfortunate confounding effect when interpreting experimental results. As an example, a researcher selects a strain from the CLiP mutant library that has a mutant gene of unknown function. The researcher compares that mutant strain to their preferred WT strain, CC‐125, in an assay to quantify ST capacity. The researcher observes a significant decrease in the mutant strain relative to the WT and thus assumes that the decrease in ST capacity is due to the mutated gene. However, the CLiP mutant library was produced in a background strain, CC‐4533, which we demonstrate here has significantly lower ST capacity than other WT strains like CC‐125 (Figure 8). Thus, the phenotypic differences between CC‐4533 and other WT strains that we document in this work may lead an unaware researcher to erroneously assign a role in ST to the mutant gene. Similarly, we document significant differences in cell size and TOC between strains (Figure S1) that could dramatically alter a comparison of two strains depending on whether a particular measurement was normalized to cell number or to total carbon content. Finally, we note that the substantial differences we observed in basal transcription (Figure 3), are likely the result of differences that have accumulated recently during propagation in the laboratory. Consequently, even comparisons between two strains with the same haplotype (Figure 3A) and minimal genomic variants between them, have the potential to be affected by transcriptional drift that may occur over time.

## EXPERIMENTAL PROCEDURES

### Strains

Eight strains of *C. reinhardtii* were used in this study: CC‐124, CC‐125, CC‐1009, CC‐1690, CC‐1691, CC‐4402, CC‐4532, and CC‐4533 (Gallaher et al., 2015), as indicated. All are available from the Chlamydomonas Resource Center at the University of Minnesota (https://www.chlamycollection.org/).

### Growth conditions: Trophic growth curves, Cu deficiency

Cultures were grown at 24°C with 160 rpm agitation in Tris‐acetate‐phosphate (TAP) or Tris‐phosphate (TP) medium (Harris, 2009) with a revised micro‐nutrient composition (Kropat et al., 2011) (Figures 1, 2). Photoheterotrophic cultures were grown in TAP medium with 70–80 μmol m^−2^ s^−1^ continuous illumination (2:1 ratio of 4100 K cool white and 3000 K warm white fluorescence bulbs). Heterotrophic (dark) cultures were grown in TAP without illumination (<5 μmol m^−2^ s^−1^). Photoautotrophic cultures were grown in TP medium with 70–80 μmol·m^−2^·s^−1^ continuous illumination (2:1 ratio of 4100 K cool white and 3000 K warm white fluorescence bulbs) and were supplied with sterile air for increased CO_2_ availability (air flow rate of 0.5–1 L min^−1^ L^−1^ culture volume). Cultures were maintained photoheterotrophically and were adapted to heterotrophic (dark) or photoautotrophic growth conditions for a period of at least two full growth curves prior to the inoculation of experimental cultures.

For the Cu‐deficiency study, replicate cultures were transferred sequentially two times from inoculation to stationary growth phase in photoheterotrophic growth medium without added Cu to generate cells acclimated to severe Cu deficiency. After two rounds of growth in media without Cu, the experimental cultures containing EDTA‐chelated Cu or not, and supplemented with 200 mM EDTA‐chelated Fe (10‐fold excess relative to standard medium (Kropat et al., 2011)) or not were inoculated at an initial density of 1–2 × 10^4^ cells·mL^−1^ and sampled for Chl content at a density of 4–5 × 10^6^ cells·mL^−1^.

Growth was monitored from inoculation to stationary phase, and the cell number was determined every 12 h (photoheterotrophic) or 24 h (heterotrophic) by manual counting (hemocytometer). The generation time was calculated from the resulting growth curves, starting when each culture reached 1 × 10^5^ cells·mL^−1^ up to the first time point of stationary phase.

### Growth conditions: Transcriptomics

Pre‐cultures of each WT strain were used to inoculate either two batches of two (CC‐124, CC‐1009, CC‐1690, CC‐1691, and CC‐4532) or one batch of four (CC‐125, CC‐4533) flasks to produce four biological replicate samples for each strain (Figures 3, 4). Flasks contained Tris‐Acetate‐Phosphate (TAP) (Gorman & Levine, 1965) medium supplemented with Kropat's trace metals (Kropat et al., 2011), and were inoculated at a concentration of 1.5 × 10^4^ cells·mL^−1^. Flasks were placed under continuous agitation on a shaking platform at 180 rpm at 25°C in a growth chamber under 50–70 μmol photons m^−2^ s^−1^ of continuous light. Light was generated by fluorescent bulbs with a ratio of two cool white bulbs (4100 K) to one warm white bulb (3000 K). Cultures were grown for approximately 3 days to reach mid‐log growth phase and collected at ~2–3 × 10^6^ cells·mL^−1^.

### Growth conditions: Three light intensities

The strains were grown under three light intensities as indicated: low light (LL, ~20 μmol photons m^−2^ s^−1^ grown photoheterotrophically in Tris‐Acetate‐Phosphate medium (TAP); Gorman & Levine, 1965), medium light (ML, ~80 μmol photons m^−2^·s^−1^ grown photoheterotrophically in TAP), and high light (HL, ~500 μmol photons m^−2^ s^−1^ grown photoautotrophically in high salt medium (HSM); Sueoka, 1960) with ambient CO_2_ under axenic conditions (Figures 5, 6, 7, 8, 9). Cells were grown at 25°C 140 rpm and collected during the log phase of growth for each experiment. The cultures were diluted 1 day before each physiological experiment. For LL and ML, samples were grown in TAP for at least 96 h before collection. For HL treatment, cells were pre‐grown in ML in TAP for at least 72 h and then transferred into HSM. Aliquots from HL‐treated samples were collected at 0, 4, 6, 7.5, 24, 48, and 144 h (6 days) HL exposure. Cells were dark‐adapted in open flasks shaking vigorously for at least 20 min before each measurement to make the PQ pool oxidized via chlororespiration (Nawrocki et al., 2015).

### RNA‐Seq library generation and analysis

Total RNA was purified using commercial kits as described previously (Dupuis et al., 2025). Poly‐adenylated mRNA was selected and used to construct libraries for sequencing on the Illumina platform following standard protocols with commercial kits from Illumina. Sequencing was performed on the Illumina HiSeq 3000 with 50 nt single‐end reads.

The resulting data were mapped to the *C. reinhardtii* CC‐4532 reference genome assembly and gene annotations (v6.1) available from Phytozome.net. Mapping was performed with STAR (Dobin et al., 2013) using the following parameters: ‐‐alignIntronMax 3000 ‐‐outSAMtype BAM SortedByCoordinate ‐‐outMultimapperOrder Random ‐‐outSAMmultNmax 1 ‐‐limitBAMsortRAM 2 000 000 000 ‐‐outSAMunmapped Within. All subsequent analyses were performed using the R statistical computing platform with analysis packages as described below. Reads were assigned to genes with the featureCounts() function of Rsubread package using the following parameters: annot.ext = annotations, isGTFAnnotationFile = ‘SAF’, useMetaFeatures = TRUE, allowMultiOverlap = TRUE, fracOverlap = 0.5, minOverlap = 30, largestOverlap = TRUE, countMultiMappingReads = TRUE, fraction = TRUE, minMQS = 0, primaryOnly = TRUE, ignoreDup = FALSE, strandSpecific = 2, genome = genome, isLongRead = FALSE, isPairedEnd = FALSE, countReadPairs = TRUE, checkFragLength = TRUE, minFragLength = 50, maxFragLength = 3000, countChimericFragments = FALSE, autosort = TRUE. Differentially expressed genes (DEGs) and normalized expression estimates in terms of fragments per kb of gene per million mapped reads (FPKMs) were calculated with the DESeq2 package (Love et al., 2014). A gene was considered a DEG between two strains if it met the following criteria: (1) >1 FPKM in at least one of the two strains, (2) an *s*‐value <0.01, and (3) >2‐fold difference between strains. PCA analysis was performed on the 1000 genes with the largest inter‐strain variance using the prcomp() function in the stats package, and plotted with the ggplot2 package. *k*‐means clustering was performed with the kmeans() function in the stats package. Gene ontology (GO) enrichment was performed with the enricher() function in the clusterProfiler package. Heatmaps were generated with the ComplexHeatmap package. The hypergeometric distribution was calculated with the phyper() function in the stats package.

### Pigment analysis

Absorption spectra were measured with a Cary 4000 UV–Vis spectrophotometer. Pigments were extracted from the cells in 80% acetone (Sigma‐Aldrich, Saint Louis, MO, USA) saturated with Na_2_CO_3_ (Porra et al., 1989) and the spectra of the extract were fitted with the spectra of individual pigments according to (Croce et al., 2002). HPLC was performed as described in (Gilmore & Yamamoto, 1991) with a few modifications: acetonitrile: methanol: 0.1 M Tris–HCl (pH 7.6) = 72: 8: 10 (v/v).

### Physiological measurements

Joliot‐type spectrophotometer JTS‐10 (Biologic, Seyssinet‐Pariset, Grenoble, France) was used to perform fluorescence measurements. The cultures were placed in a custom‐made cuvette holder and were stirred during measurements. To sufficiently induce NPQ, an LED actinic light (1500 μmol photons m^−2^·s^−1^ peaking at 630 nm (BeamBio, La Rochelle, France)) was used, which is about three times higher than the high light growth condition. Saturating pulses of >10 000 μmol photons m^−2^·s^−1^ and 180 ms pulse duration were used. Fluorescence was triggered by 10 μs flashes of white light passing through a 520 nm (10 nm FWHM) Schott filter and detected after a longpass >660 nm filter.

### NPQ measurements

After the 20‐min dark incubations to relax any quenching mechanisms acting in HL, NPQ was measured as follows. A short 5.5‐min protocol was used, in which the illumination phase (to calculate maximum NPQ) was 2.5 min after the short 10‐second dark period (when F_0_ was acquired), and the subsequent recovery period (in darkness) was 3 min. Non‐photochemical quenching (NPQ) was calculated as (*F*_m_/*F*_m_′)‐1, and the first 50‐second fluorescence signals (within the illumination) were used for maximum NPQ calculation to avoid the possible superposition of state transitions that occur on longer timescales. Maximum quantum efficiency of PSII photochemistry (*F*_v_/*F*_m_) was calculated as (*F*_m_ − *F*_0_)/F_m_. All parameters were calculated according to (Baker, 2008).

Acetic acid‐induced NPQ was estimated with a DUAL‐PAM (Walz). 1 M acetic acid was used to decrease the pH of the cell suspension to 5.5. The quenching was then reversed by adding 2 M KOH to neutralize the pH to the value prior to the acid addition to ensure quenching reversibility.

### Functional antenna size of PSII

The kinetics of fluorescence from dark‐adapted cells (PSII open centers, Q_A_ maximally oxidized) to light (PSII closed centers, Q_A_ maximally reduced) were recorded in the presence of dichlorobenzyl dimethyl urea (DCMU, 10 μM, added after the 20‐min dark incubation) by a low intensity red actinic light ~35 μmol photons m^−2^·s^−1^ peaking at 630 nm (Nawrocki et al., 2019; Tian et al., 2019). The dark acclimation was achieved via the 20‐min dark incubations as mentioned above.

### State transitions

To measure the maximal amplitude of ST, State I was induced via aerobic dark‐incubation as mentioned above, followed by subsequent illumination with red light in the presence of 10 μM DCMU that was added to the sample after the dark‐incubations. During the illumination the fluorescence signal was measured every 2 mins, and State I was considered to be complete upon achieving a stable fluorescence level (generally after ~10 ± 2 min of illumination). In turn, State II was induced by the addition of glucose (20 μM) and glucose oxidase as described in (Nawrocki et al., 2016) to establish anoxia. To measure maximum fluorescence (*F*_m_′), red actinic light with low intensity was used and *F*_m_′ was recorded every ~2 min. To calculate state transitions (%), every *F*_m_′ was normalized to the last *F*_m_′ point in state I induction, meaning that the point of *F*_m_′ was recorded before the addition of glucose and glucose oxidase. These kinetics were then plotted and the STI‐>STII transition was fitted with a monoexponential decay function (*y* = A1*exp(−*x*/*t*1) + *y*0) in OriginLab software.

### Electrochromic shift (ECS) measurements

PSI:PSII reaction center ratio was measured according to (Alric et al., 2010; Joliot & Joliot, 2005) with minor modifications (Nawrocki et al., 2016), using green Nd:YAG laser pumping a red fluorescent dye to trigger photochemistry and the signal was detected (at 520 nm) 140 μs after the laser flash.

### Sample preparations and immunoblots

Total cell protein extracts and immunoblots were performed according to (Dinc et al., 2014; Ramundo et al., 2013) with a few modifications (Nawrocki et al., 2020). 5 μg total cell protein extracts were loaded on each well. All antibodies were purchased from Agrisera (Sweden).

## AUTHOR CONTRIBUTIONS

SDG, AG, SSM, and RC conceived the experiments. XL, OV, SDG, WN, and AG performed the experiments. XL, OV, WN, SDG, and AG analyzed data. SDG, SSM, and RC wrote the manuscript with contributions from all authors.

## CONFLICT OF INTEREST

The authors declare that they have no conflicts of interest associated with this work.

## Supporting information


**Dataset S1.** DEGs with haplotypes.

**Dataset S2.** GO term enrichment.

**Dataset S3.** FPKMs all genes.

**Dataset S4.** Differential expression.

**Figure S1.** Variation in cell size and its impact on estimate of biomass.
**Figure S2.** Micronutrient content.
**Figure S3.** Transcriptomic analysis of the Chl biosynthesis pathway.
**Figure S4.** Transcriptomic analysis of the carotenoid biosynthesis pathway.
**Figure S5.** Representative immunoblots.
**Figure S6.** Changes in PSI:PSII ratio.
**Figure S7.** PSII fluorescence.
**Figure S8.** NPQ capacity under HL.
**Figure S9.** LHCSR1 versus NPQ capacity after six days of HL acclimation.
**Figure S10.** NPQ versus LHCSR3 upon HL acclimation.
**Figure S11.** Correlations of LHCSR3 and NPQ_(T)_ in the WT strains after 144 h HL treatment.
**Figure S12.** Changes in Chl and Car content during HL acclimation.
**Figure S13.** PSII antenna response.

## Data Availability

Transcriptomics data, including raw Illumina sequencing reads and normalized transcript abundance values (as FPKMs), are available from the US National Center for Biotechnology Information Gene Expression Omnibus repository at accession number GSE296941.
