# Supplementary material for: Hidden diversity: Transcriptomic and photosynthetic variation among common ‘wild type’ Chlamydomonas strains
Source: Plant J. 2025 Dec 5;124(5):e70615. doi: 10.1111/tpj.70615 (PMC12680403; doi:10.1111/tpj.70615)
Supplement: Supplementary file 5 — Figure S1. Variation in cell size and its impact on estimate of biomass. Figure S2. Micronutrient content. Figure S3. Transcriptomic analysis of the Chl biosynthesis pathway. Figure S4. Transcriptomic analysis of the carotenoid biosynthesis pathway. Figure S5. Representative immunoblots. Figure S6. Changes in PSI:PSII ratio. Figure S7. PSII fluorescence. Figure S8. NPQ capacity under HL. Figure S9. LHCSR1 versus NPQ capacity after six days of HL acclimation. Figure S10. NPQ versus LHCSR3 upon HL acclimation. Figure S11. Correlations of LHCSR3 and NPQ(T) in the WT strains after 144 h HL treatment. Figure S12. Changes in Chl and Car content during HL acclimation. Figure S13. PSII antenna response. [file TPJ-124-0-s001.pdf]

Hidden diversity: Transcriptomic and photosynthetic variation among common 'wild type' *Chlamydomonas* strains

Xin Liu, Olli Virtanen, Sean D. Gallaher, Wojciech J. Nawrocki, Anne G. Glaesener, Sabeeha S. Merchant, Roberta Croce

SUPPLEMENTAL RESULTS

Variation in macronutrient content

Cell size is another important phenotypic variable among strains, and one that can contribute to mis-interpreting molecular data presented on a per cell basis. In standard laboratory growth conditions (see methods) CC-124, CC-4532, and CC-1691 had very similar volumes of 353 ± 38, 328 ± 49, and 315 ± 42 μm^3, respectively. CC-1690 was significantly larger at 439 ± 69 μm^3 (134% of CC-4532) while CC-1009 was smaller at 227 ± 25 μm^3 (69% of CC-4532) (Figure S1a).

The biomass of a culture can be estimated based on the content of abundant elements like carbon, nitrogen, phosphorus and sulfur that are constituents of biological macromolecules. Indeed, C, N and S, are excellent proxies for biomass. On a per cell basis CC-1690 has more total organic carbon (TOC) (Figure S1b), but when normalized to cell volume, falls into the same range as for other strains (Figure S1c). Likewise, CC-1009 has a lower C content, but this is related to its smaller size. On the other hand, CC-1690 showed lower P content, both on a per cell or per volume basis while for CC-1009 the P content (119.0 ± 27.4 $\times 10^6$ atoms·μm^{-3}) is notably (2- to 3-fold) increased relative to CC-4532 (61.0 ± 15.4 $\times 10^6$ atoms·μm^{-3}) or CC-1690 (43.0 ± 8.4 $\times 10^6$ atoms·μm^{-3}). This may relate to variation in the polyphosphate content of individual genotypes, which in turn can impact other elemental profiles and aspects of metabolism, , including increased Ca content (Tsednee *et al.*, 2019; Hui *et al.*, 2022; Aksoy *et al.*, 2014). Indeed, we note that CC-1009 also has a high Ca content (Figure S2).

Exploring factors that may modulate NPQ

In the main text, we demonstrate that there is no correlation between the steady-state NPQ level and accumulation of LHCSR3. Here we attempt to identify other possible explanations for the differences in NPQ. The first candidate is a different lumenal pH. Acetic acid pH titration has been used to obtain a stable and reversible quenched state in algae without the need for illumination (Endo and Asada, 1996; Dinc *et al.*, 2016; Kosuge *et al.*, 2018; Tian *et al.*, 2019), because lowering the pH leads to protonation of the lumenal residues of LHCSRs (Bonente *et al.*, 2011; Liguori *et al.*, 2013; Ballottari *et al.*, 2016). This approach allows measurement of NPQ capacity independent of the functionality of the electron transport chain and ATPase activity. To check whether the maximum level of NPQ correlates with the amount of LHCSR3, we measured acid-induced NPQ in the cells throughout the HL treatment. No correlation was found between acetic acid-induced NPQ and the amount of LHCSR3 when comparing different strains (Figure S11). To rule out the possibility of different amounts of static quenching being present in the strains, we calculated the theoretical NPQ ($NPQ_{(T)}$) as proposed previously (Tietz *et al.*, 2017). The $NPQ_{(T)}$ parameter assumes that the entire extent of F_v/F_m decrease in the dark is due to static quenching and allows the calculation of the effective NPQ. We adopted two ways to assess $NPQ_{(T)}$: (i) using the maximum F_v/F_m of each strain measured in LL (Figure 6A and Figure S12a) and (ii) using the value of CC-1009 at 0 h HL, since this strain has the highest F_v/F_m of all WTs (Figure S12b). As with the comparison between NPQ and LHCSR3 (Figure 9C), no linear correlation was observed between $NPQ_{(T)}$ and LHCSR3 at 144 h HL with either type of $NPQ_{(T)}$ estimation (Figure S12)

SUPPLEMENTAL FIGURES

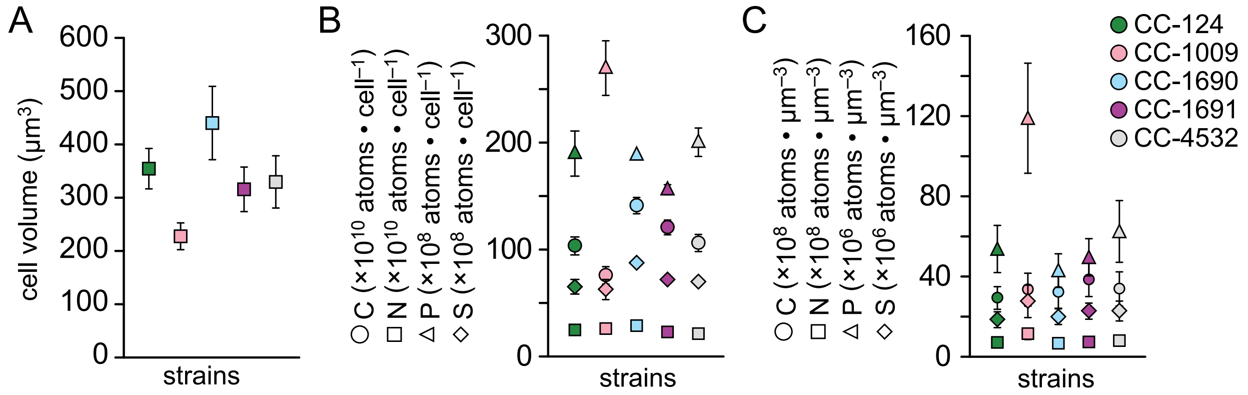

Figure S1. Variation in cell size and its impact on estimate of biomass.

Photoheterotrophically-grown cells were grown to a density of 2 $\times 10^6$ cells$\cdot$mL^{-1} and sampled for measurements. (A) The cell volume was calculated, assuming a sphere, from the median diameter measured for four independent cultures of each strain. Biomass was estimated as the elemental content on a per cell (B) or per volume (C) basis. Carbon (circle) and nitrogen (square) contents were measured by TOC-TN analysis. Phosphorous (triangle) and sulfur (diamond) content were measured by ICP-MS. The mean of four replicates is shown, error bars represent SD.

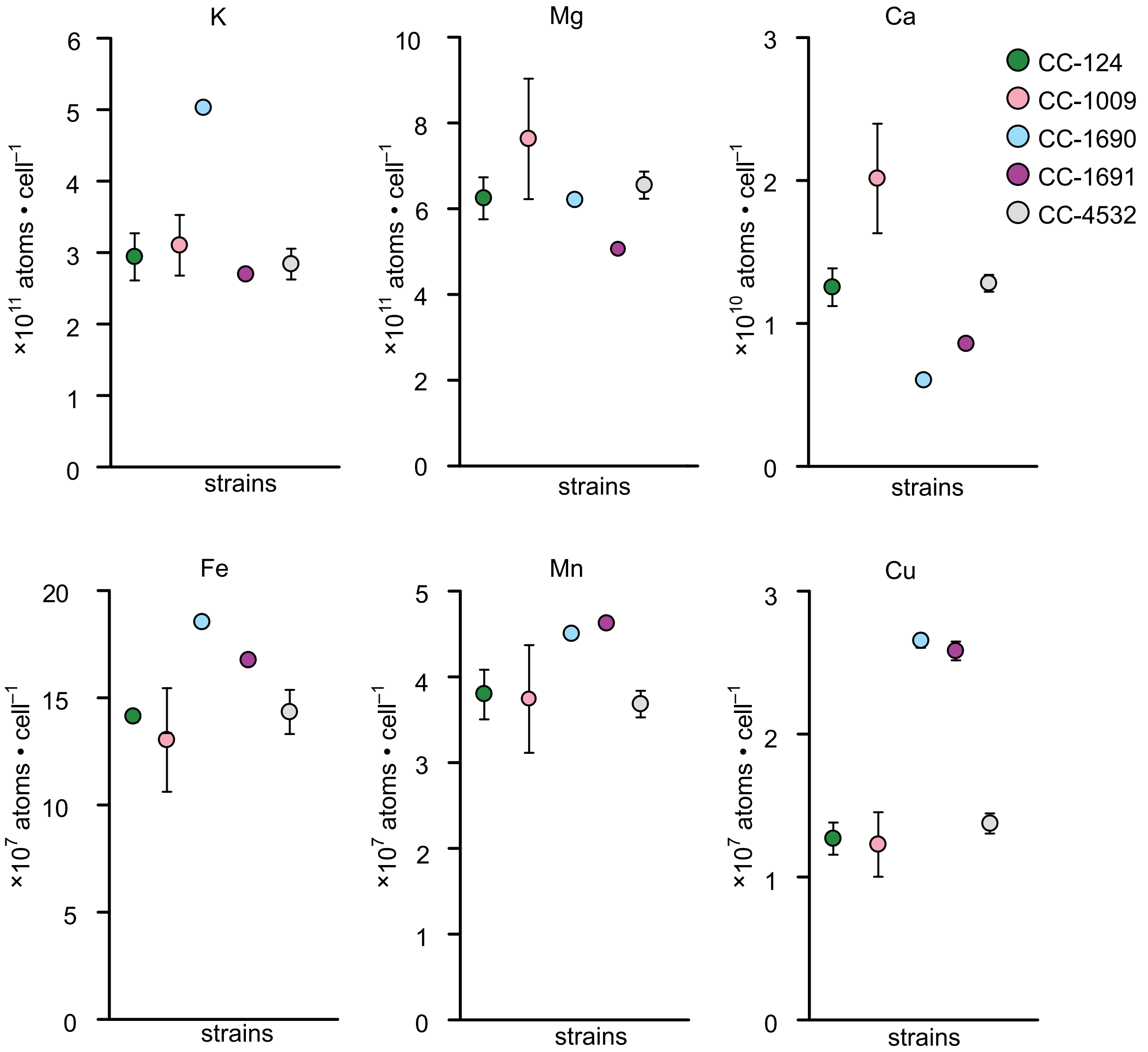

Figure S2. Micronutrient content.

Photoheterotrophic cultures were grown to a density of 2 $\times 10^6$ cells$\cdot$mL^{-1} in replete media for elemental analysis. Cellular K, Mg, Ca, Fe, Mn and Cu content were measured by ICP-MS/MS. The mean of four replicates is shown. Error bars represent SD.

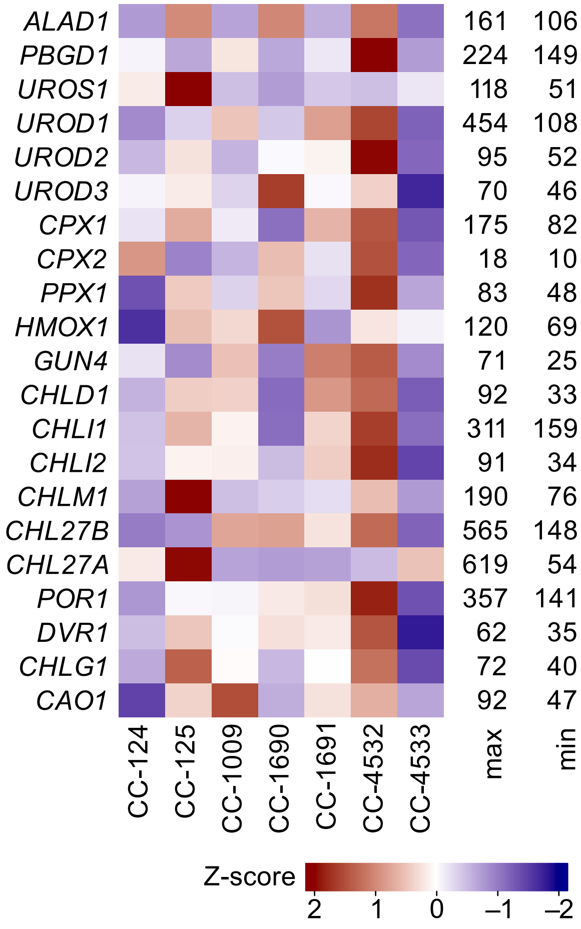

Figure S3. Transcriptomic analysis of the Chl biosynthesis pathway.

Transcript abundances were calculated for the seven indicated strains in terms of FPKMs for nucleus-encoded genes that encode components of the chlorophyll biosynthesis pathway. FPKMs were Z-score normalized and plotted as a heatmap. The maximum and minimum FPKMs for each gene are shown on the right. Gene symbols from the v6.1 gene annotations are labeled.

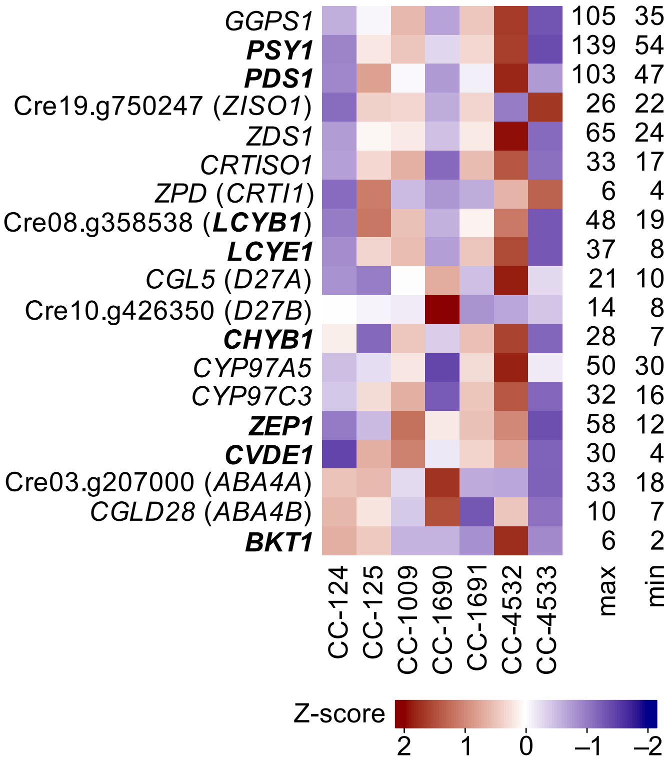

Figure S4. Transcriptomic analysis of the carotenoid biosynthesis pathway.

Transcript abundances were calculated for the seven indicated strains in terms of FPKMs for nucleus-encoded genes that are known or predicted to encode components of the carotenoid biosynthesis pathway (Goodenough, 2023). FPKMs were Z-score normalized and plotted as a heatmap. The maximum and minimum FPKMs for each gene are shown on the right. Gene symbols (if annotated) or gene identification numbers from the v6.1 gene annotations are labeled. Updated gene symbols proposed in Table 22.1 of (Goodenough, 2023) are indicated in parentheses. Symbols in bold have been experimentally confirmed.

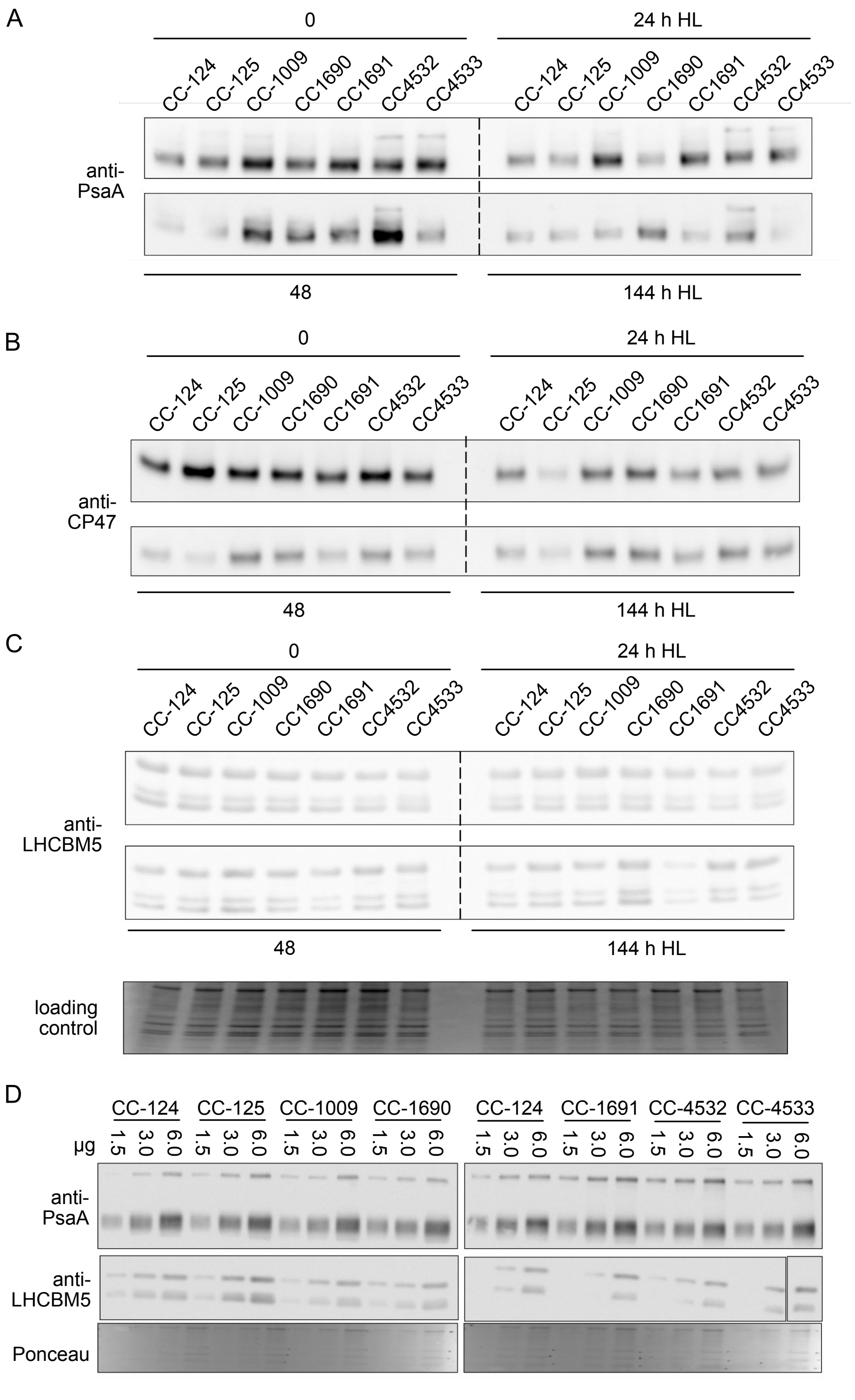

Figure S5. Representative immunoblots.

Immunoblots showing the levels of (A) PsaA, (B) CP47, and (C) LHCBM5 at different time points during HL acclimation. (D) Representative loading series (1.5, 3.0 and 6.0 µg of total protein) of anti-PsaA and anti-LHCBM5 antibodies. 5 µg of total protein extracts were loaded on each well in (A)-(C).

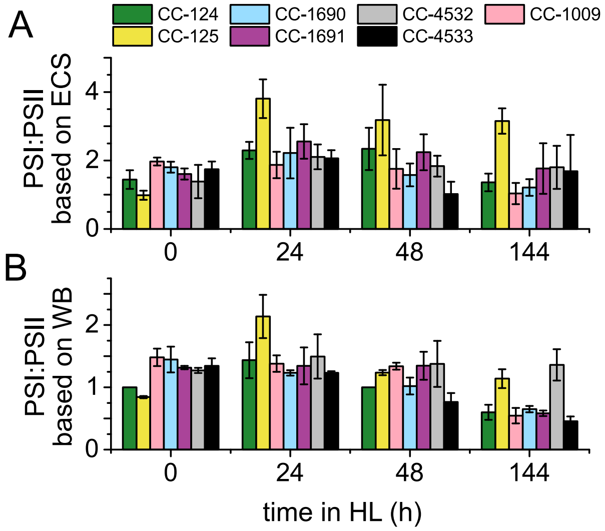

Figure S6. Changes in PSI:PSII ratio.

The functional (A) and immunoblotting-dependent (B) PSI:PSII ratios during the entire HL exposure.

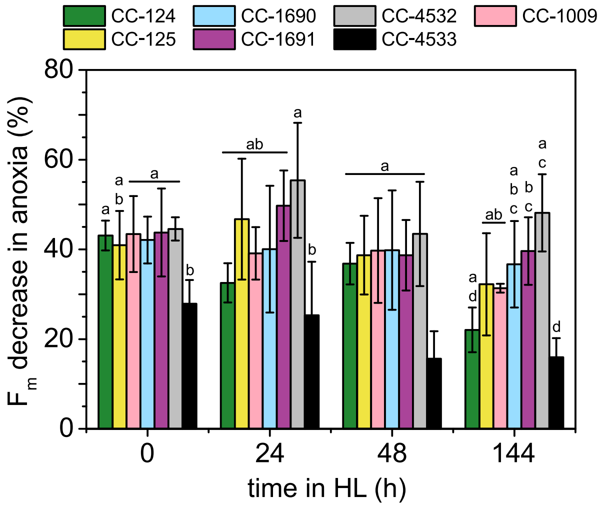

Figure S7. PSII fluorescence.

Changes in the amplitude of maximal PSII fluorescence decrease during state transitions in response to HL acclimation. Data shown are mean ± SD, n = 5 for 0 h and 144 h; n = 4 for 24 h and 48 h, n = biological replicates. Identical letters above the bars indicate a lack of statistically significant difference ($p<0.05$) between strains, examined via one-way ANOVA independently for each timepoint.

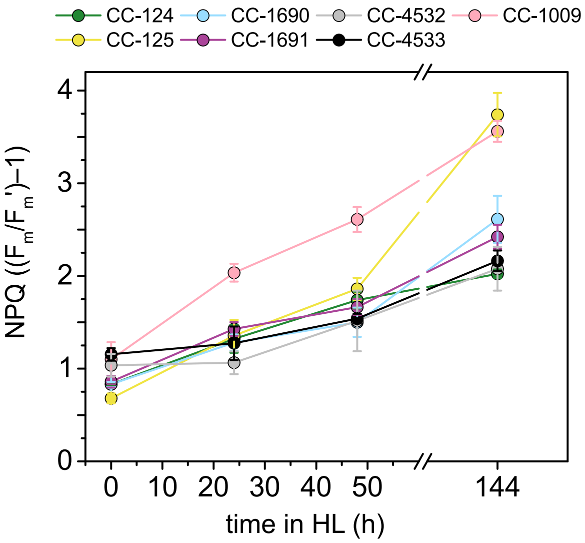

Figure S8. NPQ capacity under HL.

Cultures of the indicated strains were grown photoautotrophically in ML. At t = 0, cultures were transitioned to photoautotrophic growth in HL, and assayed for NPQ capacity. Increases in NPQ capacity are plotted for each strain over six days of HL acclimation.

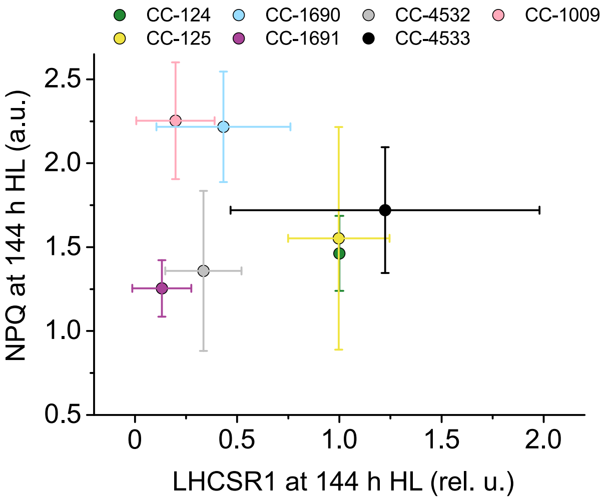

Figure S9. LHCSR1 versus NPQ capacity after six days of HL acclimation.

Immunoblots were normalized to LHCSR1 in CC-124. Both LHCSR1 quantifications and NPQ values are mean ± SD, n = 5 biological replicates.

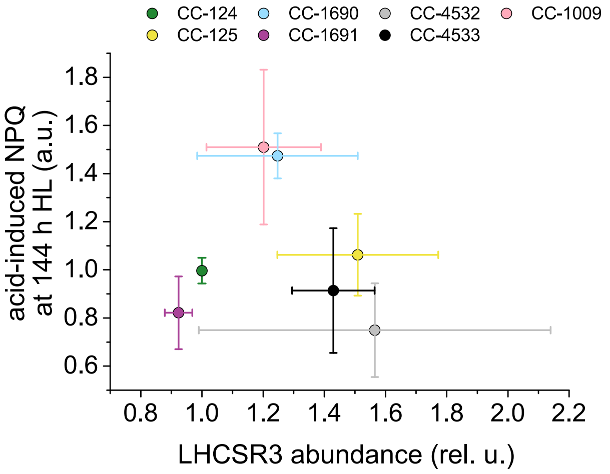

Figure S10. NPQ versus LHCSR3 upon HL acclimation.

Correlation between acetic acid-induced NPQ and LHCSR3 content after six days of HL acclimation. The data are normalized to the LHCSR3 content in CC-124 and are mean ± SD, n = 3.

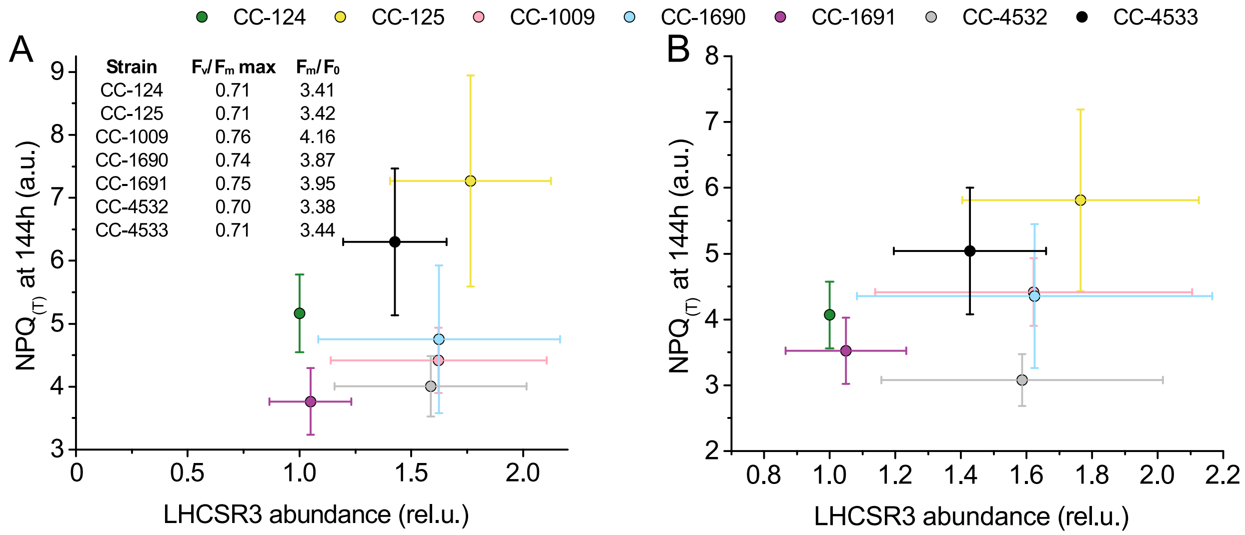

Figure S11. Correlations of LHCSR3 and $NPQ_{(T)}$ in the WT strains after 144 h HL treatment.

$NPQ_{(T)}$ was calculated with the equation: $NPQ_{corr} = (F_m/F_0 \cdot (1\text{-}F_v/F_m) \cdot F_m/F_m') - 1$, where the F_m/F_0 of each strain separately in (A) and by utilizing the highest value 4.16 of CC-1009 in (B).

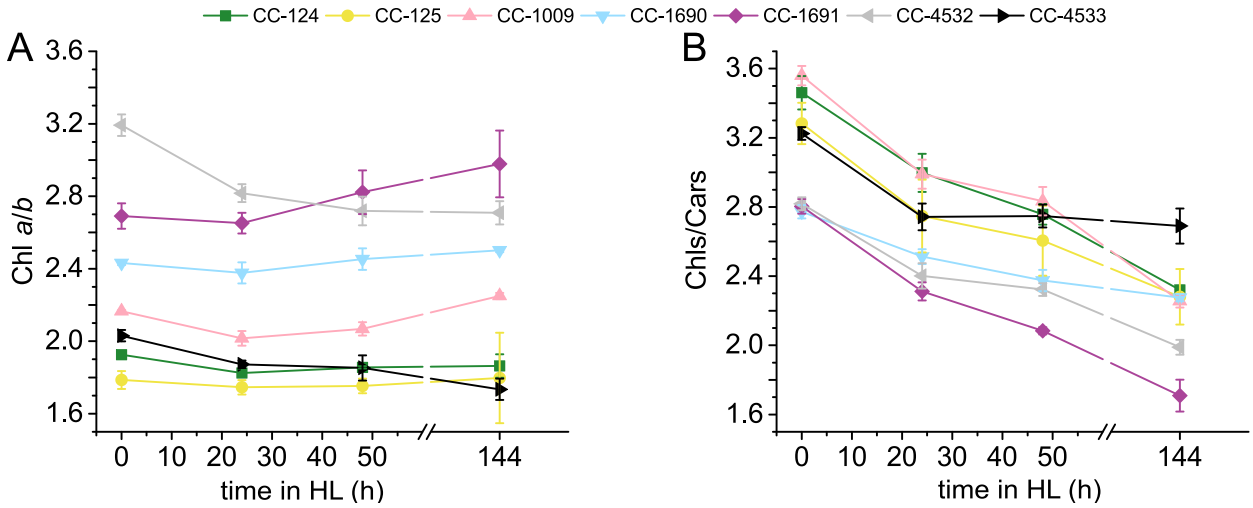

Figure S12. Changes in Chl and Car content during HL acclimation.

Changes in (A) Chl *a*/*b* ratio and (B) the Chls/Cars ratio during HL acclimation are plotted over six days of HL acclimation. Cells were pre-acclimated to photoautotrophy by growing them under moderate light in HSM for 6 generations prior to HL exposure. n = 3 biological replicates.

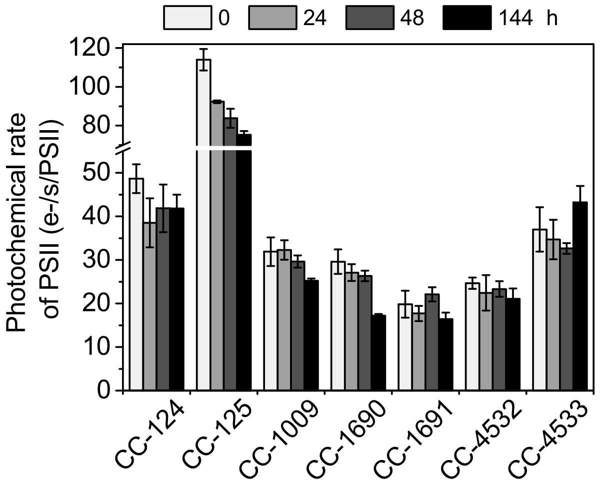

Figure S13. PSII antenna response.

Changes in functional PSII antenna in response to HL acclimation in cells pre-acclimated to photoautotrophy by growing them under ML in HSM for six generations prior to HL exposure. n = 3 biological replicates.

SUPPLEMENTAL METHODS

Estimation of cell volumes

The size distribution of healthy mid-logarithmic cells was determined by using a cell counter to estimate the median diameter of at least 500 individual cells per strain per culture. Assuming each cell approximates a sphere, the median volume of four independent cultures was calculated for each strain.

Elemental analysis by Inductively Coupled Plasma (ICP)-MS/MS

1 $\times 10^8$ cells were collected in 50-mL tubes by centrifugation (2 min, 2 500 *g*, 20°C), washed twice with 50 mL 1 mM Na_2-EDTA, pH 8.0 (to remove surface-bound metal ions), quantitatively transferred to 15-ml tubes and rinsed twice with 15 mL purified water. Pelleted cells without any remaining supernatant were carefully overlaid with 286 µL nitric acid (~70% v/v). Samples were lysed at RT for 20-24 h, followed by 1-2h at 65°C to completely dissolve the biomass, and diluted with 9.6 mL deionized water to a final nitric acid concentration of 2%. Elemental compositions were determined by ICP-MS/MS on an Agilent 8800 triple quadrupole ICP-MS/MS instrument with autosampler and standard sample introduction system by comparison to an environmental calibration standard (Agilent 5183-4688), as well as a sulfur (Inorganic Ventures CGS1) and phosphorus (Inorganic Ventures CGP1) standard, ^{89}Y (Inorganic Ventures MSY-100PPM), which is not naturally present in algae, served as an internal standard to monitor signal recovery, ion suppression and matrix effects. The concentrations of the analytes were determined in MS/MS mode, ^{24}Mg, ^{55}Mn, and ^{63}Cu were analyzed directly with helium (He) in collision mode, ^{39}K, ^{40}Ca, and ^{56}Fe were analyzed directly with hydrogen (H_2) in reaction mode, ^{31}P and ^{32}S were analyzed with oxygen (O_2) in reaction mode and detected as mass-shifted ions (Q2 = Q1 + 16). The average of four technical replicates was used for each individual biological sample, the variation between the technical replicates never exceeded 5% for an individual sample. Taking the natural abundance of isotopes into account, the total abundance of each element in each sample was automatically calculated within the Agilent MassHunter software operating the ICP-MS/MS from the single isotope detection. Total elemental content is shown in Figures S1 and S2.

Elemental analysis of Total Organic Carbon and Total Nitrogen (TOC-TN)

3 $\times 10^7$ cells were collected by centrifugation (5 min, 2 500 *g*, 4°C), washed twice with 50 mL purified water, resuspended in 3 M HCl to a final volume of 540 µL and incubated for 16h at 65°C with constant agitation. Fractions of the cell lysate were diluted to 1 $\times 10^5$ cells$\cdot$ml^{-1} with deionized water (180 µL cell lysate and 19.82 mL water) and analyzed for non-purgeable organic carbon (C) and total nitrogen (N) content using a TOC-I CSH with TNM-I unit and autosampler (Shimadzu Corp). In the instrument, a fraction of the sample (200 µL) is acidified with H_3PO_4, purged of inorganic carbon by sparging with purified air, and the remaining non-purgeable organic carbon oxidized to CO_2 at 720°C in the presence of a catalyst. The resulting CO_2 concentration was measured in technical triplicates with a non-dispersive infrared (NDIR) gas analyzer. Nitrogen oxide from the oxidized total nitrogen was analyzed in triplicates on a chemiluminescence gas analyzer. The peak area was calculated and compared to calibration curves generated from separate C and N solutions containing 25 mg C$\cdot$l^{-1} (potassium hydrogen phthalate) and 25 mg N$\cdot$l^{-1} (potassium nitrate), respectively.

SUPPLEMENTAL REFERENCES

Aksoy, M., Pootakham, W. and Grossman, A.R. (2014) Critical function of a Chlamydomonas reinhardtii putative polyphosphate polymerase subunit during nutrient deprivation. *Plant Cell*, **26**, 4214–4229.

Ballottari, M., Truong, T.B., Re, E., De, Erickson, E., Stella, G.R., Fleming, G.R., Bassi, R. and Niyogi, K.K. (2016) Identification of pH-sensing Sites in the Light Harvesting Complex Stress-related 3 Protein Essential for Triggering Non-photochemical Quenching in Chlamydomonas reinhardtii. *J Biol Chem 291, 7334-7346*.

Bonente, G., Ballottari, M., Truong, T.B., Morosinotto, T., Ahn, T.K., Fleming, G.R., Niyogi, K.K. and Bassi, R. (2011) Analysis of LhcSR3, a protein essential for feedback de-excitation in the green alga Chlamydomonas reinhardtii. *plos Biol 9, e1000577*.

Dinc, E., Tian, L., Roy, L.M., Roth, R., Goodenough, U. and Croce, R. (2016) LHCSR1 induces a fast and reversible pH-dependent fluorescence quenching in LHCII in Chlamydomonas reinhardtii cells. *Proc Natl Acad Sci U S a 113, 7673-7678*.

Endo, T. and Asada, K. (1996) Dark Induction of the Non-Photochemical Quenching of Chlorophyll Fluorescence by Acetate in Chlamydomonas reinhardtii. *Plant and Cell Physiology 37, 551-555*.

Goodenough, U. ed. (2023) The Chlamydomonas Sourcebook. In *The Chlamydomonas Sourcebook (Third Edition)*. Academic Press. Available at: https://www.sciencedirect.com/science/article/pii/B9780128224571000212 [Accessed August 19, 2025].

Hui, C., Schmollinger, S., Strenkert, D., Holbrook, K., Montgomery, H.R., Chen, S., Nelson, H.M., Weber, P.K. and Merchant, S.S. (2022) Simple steps to enable reproducibility: culture conditions affecting Chlamydomonas growth and elemental composition. *Plant J*, **111**, 995–1014.

Kosuge, K., Tokutsu, R., Kim, M., Akimoto, S., Yokono, M., Ueno, Y. and Minagawa, J. (2018) LHCSR1-dependent fluorescence quenching is mediated by excitation energy transfer from LHCII to photosystem I in Chlamydomonas reinhardtii. *Proc Natl Acad Sci U S a 115, 3722-3727*.

Liguori, N., Roy, L.M., Opacic, M., Durand, G. and Croce, R. (2013) Regulation of Light Harvesting in the Green Alga Chlamydomonas reinhardtii: The C-Terminus of LHCSR Is the Knob of a Dimmer Switch. *J Am Chem Soc 135, 18339-18342*.

Tian, L., Nawrocki, W.J., Liu, X., Polukhina, I., Stokkum, I.H.M. and Croce, R. (2019) pH dependence, kinetics and light-harvesting regulation of nonphotochemical quenching in Chlamydomonas. *Proc Natl Acad Sci U S a 116, 8320-8325*.

Tietz, S., Hall, C.C., Cruz, J.A. and Kramer, D.M. (2017) NPQ(T) : a chlorophyll fluorescence parameter for rapid estimation and imaging of non-photochemical quenching of excitons in photosystem-II-associated antenna complexes. *Plant Cell Environ 40, 1243-1255*.

Tsednee, M., Castruita, M., Salomé, P.A., et al. (2019) Manganese co-localizes with calcium and phosphorus in Chlamydomonas acidocalcisomes and is mobilized in manganese-deficient conditions. *J Biol Chem*, **294**, 17626–17641.
